# Drug prescriptions and dementia incidence: a medication-wide association study of 17000 dementia cases among half a million participants

**DOI:** 10.1136/jech-2021-217090

**Published:** 2021-10-27

**Authors:** Tim Wilkinson, Christian Schnier, Kathryn Bush, Kristiina Rannikmäe, Ronan A Lyons, Stuart McTaggart, Marion Bennie, Cathie LM Sudlow

**Affiliations:** 1 Centre for Clinical Brain Sciences, University of Edinburgh, Edinburgh, UK; 2 Usher Institute, The University of Edinburgh, Edinburgh, UK; 3 National Centre for Population Health and Wellbeing Research, Swansea University, Swansea, UK; 4 HDR UK Wales and Northern Ireland, Health Data Research UK, London, UK; 5 Public Health and Intelligence Strategic Business Unit, NHS National Services Scotland, Edinburgh, UK; 6 Strathclyde Institute of Pharmacy and Biomedical Sciences, University of Strathclyde, Glasgow, UK; 7 HDR UK Scotland, Health Data Research UK, London, UK

**Keywords:** dementia, pharmacoepidemiology, record linkage, neuroepidemiology

## Abstract

**Background:**

Previous studies have suggested that some medications may influence dementia risk. We conducted a hypothesis-generating medication-wide association study to investigate systematically the association between all prescription medications and incident dementia.

**Methods:**

We used a population-based cohort within the Secure Anonymised Information Linkage (SAIL) databank, comprising routinely-collected primary care, hospital admissions and mortality data from Wales, UK. We included all participants born after 1910 and registered with a SAIL general practice at ≤60 years old. Follow-up was from each participant’s 60th birthday to the earliest of dementia diagnosis, deregistration from a SAIL general practice, death or the end of 2018. We considered participants exposed to a medication if they received ≥1 prescription for any of 744 medications before or during follow-up. We adjusted for sex, smoking and socioeconomic status. The outcome was any all-cause dementia code in primary care, hospital or mortality data during follow-up. We used Cox regression to calculate hazard ratios and Bonferroni-corrected p values.

**Results:**

Of 551 344 participants, 16 998 (3%) developed dementia (median follow-up was 17 years for people who developed dementia, 10 years for those without dementia). Of 744 medications, 221 (30%) were associated with dementia. Of these, 217 (98%) were associated with increased dementia incidence, many clustering around certain indications. Four medications (all vaccines) were associated with a lower dementia incidence.

**Conclusions:**

Almost a third of medications were associated with dementia. The clustering of many drugs around certain indications may provide insights into early manifestations of dementia. We encourage further investigation of hypotheses generated by these results.

## Introduction

Dementia is a major global health challenge.^1^ There were an estimated 47 million people living with dementia in 2015, and the global prevalence is estimated to rise to 131 million by 2050.^2^ In the absence of disease-modifying therapies, prevention of dementia by addressing modifiable risk factors is a key goal.^3^ Recent studies have suggested that some medications, such as anticonvulsants^4^ and anticholinergic drugs,^5 6^ may be associated with increased risk of dementia, raising the possibility that avoiding these medications could reduce dementia incidence. The availability of large, population-based healthcare datasets, coupled with increasing computational capacity, provides the opportunity to explore systematically the association between medications and diseases, enabling the discovery of previously unknown associations and reducing the likelihood of selective analysis and reporting.^7^

Environment-wide association studies (EWAS) were developed to be analogous to genome-wide association studies, allowing a hypothesis-free approach to discovering new environmental risk factors or predictors of disease, while minimising reporting bias and controlling for the rate of false positives.^8 9^ Medication-wide association studies, a type of EWAS in which only medications are evaluated, provide a hypothesis-generating approach to studying the relationship between drugs and disease.^7 10^

We used a population-based, electronic cohort (e-cohort) to conduct a hypothesis-generating medication-wide association study to investigate the association between prescription drugs and dementia during the follow-up period.

## Methods

### Study design

We conducted a medication-wide association study using a population-based, retrospective cohort composed from routinely-collected healthcare data.

### Datasets

The Secure Anonymised Information Linkage databank (SAIL) contains individual-level, linked routinely-collected healthcare datasets for the population of Wales, UK. Currently, SAIL includes primary care data for approximately 80% of the population of Wales. Included participants are representative of the entire Welsh population with regards to age, sex and deprivation.^11^ The SAIL Dementia e-cohort (SAIL-DeC) consists of derived variables from coded, linked primary care, hospital admissions, mortality and deprivation datasets for a large subset of SAIL participants. In the UK, diagnoses within hospital admissions and mortality data are coded using the International Classification of Diseases version 10 (ICD-10) system.^12^ Primary care data in Wales are coded using the Read Version 2 (Read V2) system.^13 14^ The creation of the SAIL-DeC resource is outlined in detail elsewhere.^11^ Briefly, SAIL-DeC contains all participants within the SAIL Databank who were born between 1 January 1900 and 1 January 1958 and for whom primary care data are available (n=1.2 million). The cohort data dictionary and ICD-10 and Read V2 code lists used to derive the variables within SAIL-DeC are available at https://datashareedacuk/handle/10283/3268. This study was approved by the SAIL Information Governance Review Panel (application number 0837), which includes representatives from the Research Ethics Service and the public. Participants can opt-out of having their anonymised data provided to SAIL by making an enquiry to the relevant data provider.

### Study population and follow-up

**Figure 1:**
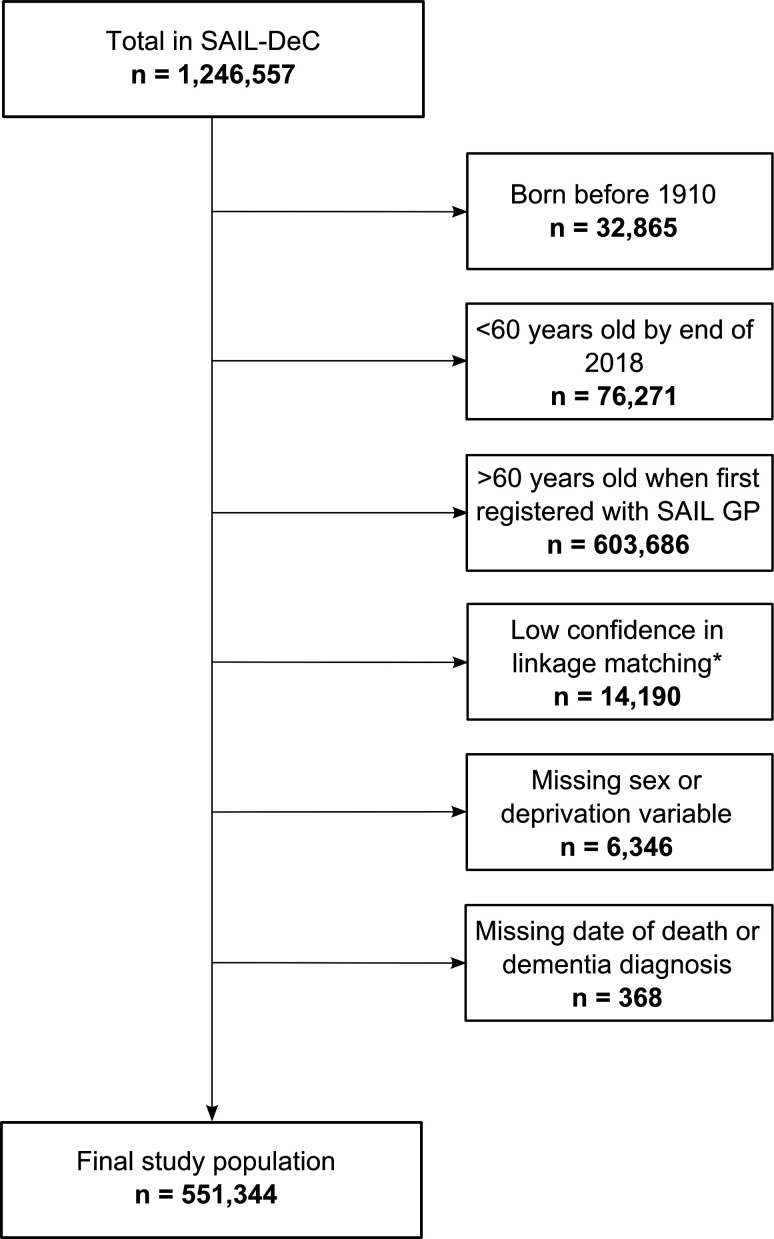
Study flow diagram. Categories are not mutually exclusive. *Confidence in linkage to any routinely-collected dataset <95%. GP, general practice; SAIL, Secure Anonymised Information Linkage databank.

We included all SAIL-DeC participants born after 1910 and first registered with a SAIL general practice when aged ≤60 years. We excluded anyone with a dementia code in primary care, hospital admissions or mortality data before their 60th birthday and those with missing month and year of birth or deprivation fields (figure 1). Follow-up for all participants started on the week of their 60th birthday and ended at the earliest of dementia diagnosis, end of registration with a SAIL general practice, death or end of 2018.

### Exposures

Prescription information in SAIL is contained within the five-byte Read V2-coded primary care data. The first three digits of the code define the drug name, with the remaining two digits describing the strength and formulation. The Read V2 system maps directly onto the hierarchical system within the British National Formulary (BNF, table 1).^15^ The BNF contains information on the indications, contraindications, side effects and doses for prescription medications in the UK. We grouped the medications data into 2626 drugs, based on the first three digits of the Read code (online supplemental material).

10.1136/jech-2021-217090.supp1 Supplementary data

**Table 1:** British National Formulary (BNF) chapters and corresponding Read V2 codes

BNF chapter	Category	Read code
1	Gastrointestinal system	a….
2	Cardiovascular system	b….
3	Respiratory system	c….
4	Central nervous system	d….
5	Infections	e….
6	Endocrine system	f….
7	Obstetrics, gynaecology and urinary-tract disorders	g….
8	Malignant disease and immunosuppression	h….
9	Nutrition and blood	i….
10	Musculoskeletal and joint diseases	j….
11	Eye	k….
12	Ear, nose and oropharynx	l….
13	Skin	m….
14	Immunological products and vaccines	n….
15	Anaesthesia	o….

The BNF is divided into chapters, each consisting of multiple sections. Read drug codes (version 2) are five digits, with the first letter indicating the corresponding BNF chapter. Medications in chapter 15 (anaesthesia) were not included in this study, as these drugs are not prescribed in primary care.

We included any drug that was prescribed to ≥1000 study participants. Participants were considered ‘exposed’ to a drug from the date of a first prescription for that medication, or from the study start date for that participant (the week of their 60th birthday) if the first prescription occurred before the age of 60. We excluded any prescriptions from the analysis that first appeared in the month before a participant’s dementia diagnosis.

### Outcome

The outcome was defined as the first all-cause dementia code in any of primary care, hospital admissions or mortality datasets, using a validated code list (online supplemental material).^16^ For patients who also received a prescription for a drug used to treat dementia (donepezil, rivastigmine, galantamine or memantine), the date of dementia diagnosis was taken as the earliest of the date of first prescription of that drug or the date of the first dementia code in any dataset. We excluded patients with a dementia drug code but no diagnostic code from the analysis, due to uncertainty about whether they represented cases or non-cases.

### Statistical analysis

We derived descriptive statistics for the study population, subdivided into those who developed dementia during follow-up (cases) or not (non-cases).

We divided the study population into derivation and validation cohorts (1:1 random allocation based on 1909 geographical household areas (Lower Layer Super Output Areas)). We used a Cox proportional hazards model, with age used as the time variable. We adjusted for sex, socioeconomic status (quintiles) and smoking status (ever- vs never-smokers). Covariate definitions are outlined in detail in the SAIL-DeC cohort profile.^11^

There is no single established method of adjusting for multiple testing in EWAS analyses,^7–9^ with a balance needing to be struck between minimising type I error (false positive results) and avoiding very large type II error (false negative results). We therefore employed two different methods. First, we used a Bonferroni correction (0.05 divided by number of drugs studied) in both the derivation and validation datasets, referring to any medications that passed this stringent threshold in each dataset as being ‘associated’ with dementia. Second, we used the Benjamini-Hochberg procedure in the derivation dataset to establish a False Discovery Rate (FDR) of 10%. For any medications passing this FDR threshold, we used a further threshold of p<0.05 in the validation dataset, referring to medications passing this as being ‘tentatively associated’ with dementia. For the purposes of reporting, we then combined the derivation and validation cohorts to produce hazard ratios (HRs) and p values for all medications.

We also conducted sensitivity analyses, using Alzheimer’s disease subtype codes (rather than all-cause dementia) as the outcome, and, to investigate the potential for reverse causation, only including medications prescribed for the first time ≥5 or ≥10 years before dementia diagnosis. For each, we plotted the log HR from the sensitivity analysis against the original log HR.

We conducted statistical analyses using R (www.r-project.org).

### Evaluating the results

One reason a medication may be associated with dementia is because it is prescribed for a condition that is itself associated with dementia (confounding by indication). Two clinicians (TW and KB) screened all associated medications, highlighting those that are commonly prescribed in one of five situations (‘Group 1’): neurodegenerative diseases, cardiovascular disease, diabetes, depression and symptoms or complications of dementia (eg, antipsychotics or nutritional supplements). This list was not designed to include all conditions that are associated with dementia, but rather to provide a framework to aid interpretation and reporting of the results.

For each of the remaining associated medications (‘Group 2’), we compared the HRs to those of all other drugs within the same BNF section to look for patterns and generate hypotheses. We considered whether HRs of medications in the same section were similar, even if small sample sizes precluded some from meeting the threshold for significance.

## Results

### Study characteristics

A total of 551 344 participants met the eligibility criteria (figure 1), resulting in 5 826 209 person-years of follow-up. Of these, 16 998 (3.1%) developed dementia during follow-up. Median follow-up time was 17.0 years for those who developed dementia and 9.7 years for those who did not receive a dementia diagnosis. Characteristics of the study population are shown in table 2.

**Table 2:** Descriptive statistics for full cohort (derivation and validation populations combined)

	Full cohort	
	Diagnosed with dementia	Not diagnosed with dementia*
Total participants	16 998	534 346
Female (%)	8903 (52)	267 340 (50)
Total follow-up time (person-years)	279 720	5 546 489
Median follow-up time (years)	17.0	9.7
Median year at start of follow-up†	1998	2007
Deprivation quintile (%)		
1 – Most deprived	3769 (22)	95 381 (18)
2	3106 (18)	98 448 (18)
3	3348 (20)	113 807 (21)
4	3048 (18)	105 032 (20)
5 – Least deprived	3727 (22)	121 678 (23)
Polypharmacy (%)	2602 (15)	79 801 (15)
Ever smoker (%)	12 207 (72)	353 090 (66)

*Did not develop dementia by the end of follow-up. Smoking was defined as being an ever- versus never-smoker. Polypharmacy was defined as being prescribed ≥10 different drugs during the first year of the observation period (from age 60–61 years).

†Median year at age 60 years.

### All medications

**Figure 2:**
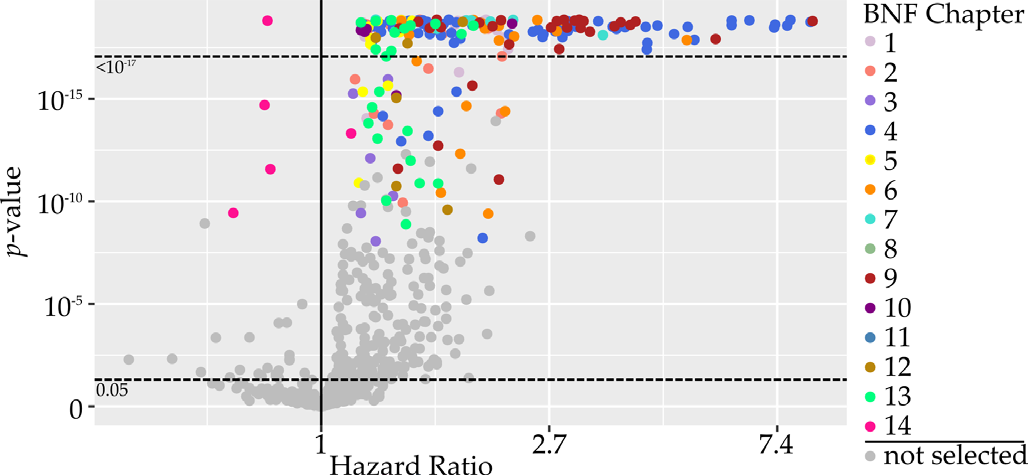
Volcano plot demonstrating the hazard ratio (HR) and p value for the association between all medications and incident dementia. Each marker represents a single medication. Grey markers represent medications that did not pass the Bonferroni-corrected threshold of significance in both derivation and validation cohorts. Coloured markers represent medications that were found to be significantly associated with dementia. Each colour represents a different British National Formulary section. HRs are on a log scale and are from the entire study population (derivation and validation cohorts combined). All markers above the upper horizontal dotted line had p values <10^−17^. The lower horizontal dotted line represents p=0.05, for reference.

A total of 744 (28%) medications were included in the analysis. With Bonferroni correction, 245 medications passed the threshold of significance in the derivation dataset. Of these, 24 did not meet the significance threshold in the validation dataset, leaving 221 (29.7%) medications ‘associated’ with dementia (figure 2). A further 100 medications were ‘tentatively associated’. Of the 221 medications associated with dementia, 217 (98%) were associated with a higher dementia incidence. Only four (2%) medications (all vaccines) were associated with a lower dementia incidence. The full results table for all 744 medications included in the analysis is available at https://datashare.ed.ac.uk/handle/10283/3792. From the 221 medications associated with dementia, 107 were indicated for one of the five situations where we predicted a priori that there could be confounding by indication (Group 1), leaving 114 medications in Group 2.

### Group 1: Medications expected a priori to be associated with dementia

Many medications indicated for neurodegenerative diseases, cardiovascular disease, diabetes, depression and symptoms or complications of dementia were associated with dementia (online supplemental material).

### Group 2: Medications expected a priori to be associated with dementia

#### BNF chapter 1: Gastrointestinal system

##### 1.1 Dyspepsia and gastro-oesophageal reflux disease

Magnesium salts, ‘compound proprietary antacids D-L’ and ‘compound proprietary antacids M-Z’ were all associated with dementia (HR range 1.22–1.63). Other drugs within this BNF section had similar HRs (HR range 1.14–1.51), suggesting a similar effect size, but with smaller numbers of exposed dementia cases.

##### 1.3 Antisecretory drugs and mucosal protectants

Ranitidine (H2 antagonist), omeprazole and lansoprazole (proton pump inhibitors) were all associated with dementia with similar HRs (HR range 1.19–1.21), while pantoprazole was tentatively associated with a similar effect size (HR 1.20).

##### 1.4 Acute diarrhoea

Loperamide was associated with dementia (HR 1.42), while the other drugs in this section were tentatively associated (diphenoxylate hydrochloride: HR 1.27; codeine phosphate for gastrointestinal tract use: HR 1.34).

##### 1.5 Laxatives

Almost all medications in this section were associated with dementia (HRs 1.39–2.27).

#### BNF chapter 4: Central nervous system

##### 4.1 Hypnotics and anxiolytics

All but one medication in this section were either associated or tentatively associated with dementia. HRs ranged from 1.27 (hydroxyzine) to 3.37 (lorazepam). In addition to benzodiazepines, other drugs for insomnia (eg, zopiclone, zolpidem and melatonin) were associated with dementia.

##### 4.6 Drugs used in nausea and vertigo

All medications in this section were associated with dementia (HR range 1.20–2.88), except ondansetron which had a similar HR (1.44) but only small numbers of exposed dementia cases.

##### 4.7 Analgesics

Apart from drugs indicated for migraine, almost all medications in this section were associated or tentatively associated with dementia (HR range 1.22–1.89). Most were opiate drugs, although medications with alternative mechanisms of action such as paracetamol and nefopam were also associated or tentatively associated.

##### 4.8 Antiepileptic drugs

All anticonvulsant drugs were associated with dementia, apart from topiramate which was tentatively associated. Effect sizes were generally large (HR range 1.53–4.99).

##### 4.10 Drugs used in substance dependence

Acamprosate calcium, used to treat alcohol dependence, was associated with dementia with a large effect size (HR 4.15). Nicotine replacement therapies were associated or tentatively associated with dementia (HR range 1.31–1.37). Bupropion and varenicline, used as smoking cessation aids, were not associated.

#### BNF chapter 5: Infections

##### 5.4 Antimalarials

Quinine, originally an antimalarial but much more widely used for leg cramps, was associated with a higher dementia risk (HR 1.23). By contrast, mefloquine hydrochloride and ‘atovaquone and proguanil hydrochloride’ were tentatively associated with a lower likelihood of developing dementia (HR range 0.60–0.63). Other antimalarials also had HRs <1, but with few cases exposed.

#### BNF chapter 7: Obstetrics, gynaecology and urinary-tract disorders

##### 7.4 Drugs for genito-urinary disorders

Tamsulosin, oxybutynin, tolterodine L-tartrate, trospium chloride, solifenacin, duloxetine and ‘catheter patency solutions’ were all associated with dementia (HR range 1.38–3.44), while flavoxate hydrochloride, fesoterodine fumarate and mirabegron were tentatively associated (HR range 1.71–2.15). Only tadalafil was tentatively associated with lower dementia incidence (HR 0.87). Of note, many of the drugs in this section have anticholinergic activity, but those without, such as mirabegron, tamsulosin and ‘catheter patency solutions’ also had HRs >1.

#### BNF chapter 14: Vaccines and antisera

Four vaccines were associated with a lower risk of dementia: hepatitis A (HR 0.78), typhoid (HR 0.80), hepatitis A and typhoid combined (HR 0.68), and diphtheria (HR 0.79). Varicella zoster vaccine was tentatively associated (HR 0.73). The influenza vaccine, which is routinely offered to patients with chronic health conditions and all people ≥65 years of age in the UK, was the only vaccine associated with a higher dementia risk (HR 1.11).

### Sensitivity analyses

**Figure 3:**
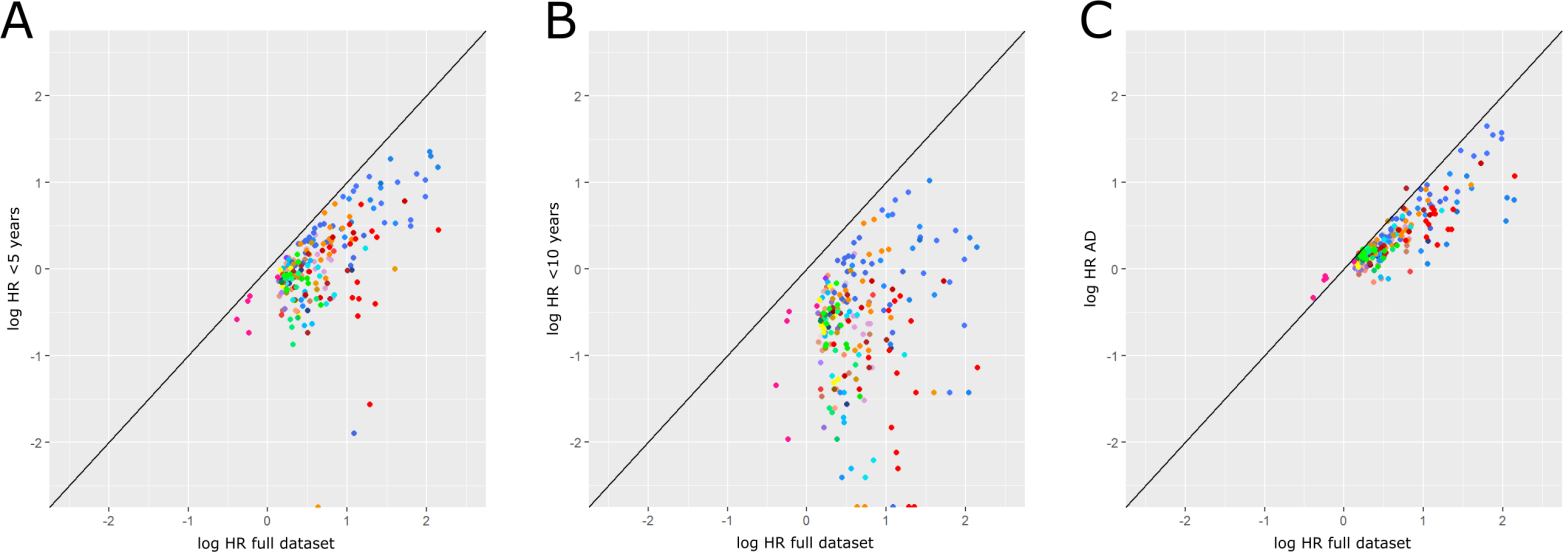
Sensitivity analyses. (A) Exclusion of drugs prescribed <5 years before dementia diagnosis, (B) exclusion of drugs prescribed <10 years before dementia diagnosis, (C) Alzheimer’s disease (AD) subtype cases only (compared with all-cause dementia). Log hazard ratio (log HR) from the sensitivity analysis plotted against the original log HR from the full dataset. Each coloured marker represents a medication that was found to be associated with dementia. Each colour reflects a different British National Formulary chapter and section. Where markers remain on the diagonal line, the log HR in the sensitivity analysis is the same as in the original analysis. If the markers are below the diagonal line, the log HR is lower in the sensitivity analysis compared with the original analysis. For markers above the line, the log HR in the sensitivity analysis is greater than that of the original analysis.

In general, when participants who first received a drug <5 and<10 years before a dementia diagnosis were excluded, HRs were lower (figure 3). Of the 114 Group 2 drugs associated with dementia, only eight still had a HR >1 when exposure was defined as first prescription ≥10 years before dementia diagnosis: dantron (used as a laxative in palliative care), nitrazepam, acamprosate (used in alcohol dependence), and five anticonvulsants (carbamazepine, phenytoin, sodium valproate, lamotrigine and primidone).

Of the four vaccines associated with a lower dementia risk, HRs were attenuated when only participants first prescribed them ≥5 and ≥10 years before dementia diagnosis were considered exposed.

There was variability in whether the HRs remained similar when only Alzheimer’s disease subtype codes were used to define the outcome (figure 3). From the Group 2 medications that were associated with dementia, 107/114 (93.9%) still had HRs >1 when Alzheimer’s disease was the outcome.

## Discussion

We conducted a hypothesis-generating medication-wide association study, which found many medications to be associated with dementia, even after strict correction for multiple testing. The vast majority of these were associated with a higher dementia incidence. Many associations diminished when only medications prescribed >5 and>10 years before dementia diagnosis were included.

### Medications associated with a lower dementia incidence

The four medications associated with a lower dementia incidence were all vaccines: hepatitis A, typhoid, hepatitis A and typhoid combined, and diphtheria. Previous studies of vaccines and dementia risk have led to hypotheses that immunological changes, modified by vaccination, may protect against dementia.^17–19^ However, the vaccines associated with a lower dementia incidence in our study were all travel vaccines, with similar HRs to several antimalarial drugs. This suggests that the observed association may be due to foreign travel, rather than a direct effect of the vaccines. The association could be due to reverse causation (ie, people with subclinical dementia pathology are less likely to travel), confounding by education or residual confounding by socioeconomic status.

### Medications associated with a higher dementia incidence

Many drugs were associated with a higher dementia incidence. As expected, many Group 1 medications were associated with dementia. Rather than exclude these medications from the analysis, we included all prescription medications in case they revealed unexpected and potentially informative findings. One example of this is lithium, a drug usually prescribed for mania and/or bipolar affective disorder, which had a high HR (3.59), possibly reflecting an association between bipolar affective disorder and dementia.^20^

Other medications associated with dementia appeared to group around certain indications, such as gastro-oesophageal reflux disease, altered bowel habit, lower urinary tract symptoms, anxiety, sleep disturbance, pain and vertigo/nausea. HRs were often similar among drugs for the same indication, even if the mechanisms of action were different.

### Reverse causation

Alzheimer’s disease and most other dementias are believed to have a long pre-clinical period, during which pathology accumulates before symptoms become apparent.^21 22^ This makes epidemiological studies of dementia prone to reverse causality, meaning subclinical dementia is causing the association with a given ‘risk factor’. Several studies have revealed changes in the magnitude or direction of associations between particular risk factors and dementia with differing follow-up times.^23–25^ To explore this issue, we conducted a sensitivity analysis in which we excluded drugs prescribed for the first time <5 and <10 years before a dementia diagnosis. Almost all HRs fell during this sensitivity analysis, with very few drugs first prescribed ≥10 years before diagnosis still being associated with a higher dementia incidence.

### Hypotheses

There are multiple potential interpretations for why some medications may be associated with dementia. We have made the full results publicly available in the hope that other researchers will use the outputs from this study to generate their own hypotheses before exploring them in external datasets.

Reverse causality is one plausible interpretation for many of the observed associations. The clustering of many of these drugs around certain indications, such as gastro-oesophageal reflux disease, altered bowel habit, lower urinary tract symptoms, anxiety, sleep disturbance, pain and vertigo or nausea, suggests that these symptoms are common in the years before dementia diagnosis. This raises the possibility of a non-cognitive syndrome in dementia, akin to that seen with the non-motor symptoms of Parkinson’s disease.^26^

### Strengths and limitations

This study benefits from a systematic approach, allowing us to identify novel associations, compare drugs with similar indications but different mechanisms of action, and avoid selective reporting and publication bias. By using a population-based cohort we have maximised the generalisability of our findings to the wider population. The long follow-up times available for some individuals allowed us to conduct sensitivity analyses to explore the possibility of reverse causation.

There are several limitations. By defining participants as ‘exposed’ to a medication if they were prescribed the drug at least once, we will have misclassified some participants as being exposed when they may never have taken the drug. We could have created an algorithm that included multiple prescriptions to minimise this risk, but this would have excluded single-use medications such as vaccines. Similarly, we did not investigate dose–response relationships, as this would be too complex for the number of drugs studied. We anticipate that researchers seeking to further investigate some of these findings will wish to explore the role of single versus multiple prescriptions and include dose–response analyses in future studies. We only adjusted for a limited number of potential confounders. For example, level of education is not available within UK routinely-collected healthcare data, so we could not adjust for this. Routinely-collected healthcare data do not identify dementia with perfect accuracy. Although the positive predictive value (the proportion of people with a dementia code who do have dementia) of using these datasets to identify dementia cases is generally high,^16^ the sensitivity (proportion of true dementia cases identified as such) is not known.^27^ However, there is evidence that people with dementia who are identified in routinely-collected data as such may systematically differ from those not identified, suggesting the potential for ascertainment bias.^28^ It is likely that patients who were prescribed certain medications were more likely to interact with various healthcare services, which could in turn have made them more likely to be subsequently diagnosed with dementia. For the primary analysis we used ‘all-cause dementia’ as an outcome, which encompasses different diseases. We chose this because the accuracy of subtype dementia codes is known to be lower,^16^ and in older age many dementias are of ‘mixed’ pathology.^29^ Lastly, this study can only be hypothesis-generating, and further studies are required to investigate interesting associations in detail.

## Conclusion

Many prescription medications are associated with a higher dementia incidence. The clustering of some drugs around certain indications may provide insights into the early stages of dementia. We encourage researchers to use these results to generate hypotheses for testing in external datasets.

**What is already known on this subject**

- Understanding and addressing modifiable risk factors is crucial for reducing dementia incidence. Previous studies have suggested that some medications may influence dementia risk. However, these associations have not been studied systematically.

**What this study adds**

- We found that almost a third of medications were associated with dementia. Drugs with differing mechanisms of action but the same indication appeared to have similar effect sizes, suggesting that many associations may be due to reverse causation as opposed to a direct causal effect. We have made our results publicly available with the intention that others may generate their own hypotheses based on these results.

## Data Availability

Data are available in a public, open access repository. Aggregated results are available in a public, open access respository. Raw data are available following application to the SAIL databank and approval from the Information Governance Review Panel.
