# Supplementary material for: Drug prescriptions and dementia incidence: a medication-wide association study of 17000 dementia cases among half a million participants
Source: J Epidemiol Community Health. 2021 Oct 27;76(3):223–9. doi: 10.1136/jech-2021-217090 (PMC8862053; doi:10.1136/jech-2021-217090)
Supplement: Supplementary data [file jech-2021-217090supp001.pdf]

Supplementary Material

Contents

Description	Page
Read version 2 medication codes	2
Codes used to identify all-cause dementia and Alzheimer's disease cases	61
Supplementary Results: Medications predicted a priori to be associated with dementia (Group 1)	65

Wilkinson T, *et al. J Epidemiol Community Health* 2022; 76:223–229. doi: 10.1136/jech-2021-217090

Read version 2 medication codes

Read code	Medication
a....	GASTRO-INTESTINAL DRUGS
a1…	OFFICIAL/SIMPLE ANTACIDS
a11..	ALUMINIUM HYDROXIDE [ANTACID]
a12..	MAGNESIUM SALTS-ANTACID
a13..	SODIUM BICARBONATE [ANTACID]
a14..	SODIUM CITRATE [ANTACID]
a2...	COMPOUND ANTACIDS
a21..	GENERIC ANTACIDS
a22..	COMPOUND PROPRIETARY ANTACIDS A-C
a23..	COMPOUND PROPRIETARY ANTACIDS D-L
a24..	COMPOUND PROPRIETARY ANTACIDS M-Z
a3...	CALCIUM/BISMUTH ANTACIDS
a31..	CALCIUM/BISMUTH ANTACIDS A-Z
a4...	ANTISPASMODICS/GASTRO-INTESTIN TRACT MOTILITY ALTERING DRUGS
a41..	ATROPINE SULPHATE [ANTISPASMODIC]
a42..	*ATROPINE METHONITRATE
a43..	*AMBUTONIUM BROMIDE
a44..	BELLADONNA ALKALOIDS
a45..	DICYCLOVERINE HYDROCHLORIDE
a46..	GLYCOPYRRONIUM BROMIDE [ANTISPASMODIC]
a47..	HYOSCINE BUTYLBROMIDE
a48..	MEPENZOLATE BROMIDE
a49..	PIPENZOLATE BROMIDE
a4a..	*PIPERIDOLATE HYDROCHLORIDE
a4b..	*POLDINE METHYLSULPHATE
a4c..	PROPANTHELINE BROMIDE [ANTISPASMODIC]
a4d..	ALVERINE CITRATE
a4e..	MEBEVERINE HYDROCHLORIDE
a4f..	PEPPERMINT OIL
a4g..	DOMPERIDONE(MOTILITY) [see chapter d for preparations]
a4h..	METOCLOPRAMIDE(MOTILITY) [see chapter d for preparations]
a4i..	LINACLOTIDE
a5...	COMPOUND ANTISPASMODICS
a51..	COMPOUND ANTISPASMODICS A-Z
a6...	ULCER-HEALING DRUGS
a61..	CIMETIDINE
a62..	RANITIDINE
a63..	PIRENZEPINE
a64..	BISMUTH CHELATE
a65..	SUCRALFATE
a66..	CARBENOXOLONE SODIUM [GASTRO-INTESTINAL USE]
a67..	LIQUORICE DEGLYCYRRHIZINISED

Wilkinson T, *et al. J Epidemiol Community Health* 2022; 76:223–229. doi: 10.1136/jech-2021-217090

a68..	FAMOTIDINE
a69..	NIZATIDINE
a6a..	MISOPROSTOL
a6b..	OMEPRAZOLE
a6c..	LANSOPRAZOLE
a6d..	RANITIDINE BISMUTH CITRATE
a6e..	PANTOPRAZOLE
a6f..	RABEPRAZOLE SODIUM
a6g..	COMBINATION ULCER HEALING DRUGS
a6h..	ESOMEPRAZOLE
a7...	ANTIDIARRHOEAL ADSORBENT MIXTURE
a71..	KAOLIN, LIGHT
a72..	*CERATONIA
a73..	CHALK
a75..	METHYLCELLULOSE [ANTIDIARRHOEAL]
a76..	*STERCULIA [ANTIDIARRHOEAL]
a8...	ANTIDIARRHOEAL-MOTILITY REDUCTION
a81..	CODEINE PHOSPHATE [GASTRO-INTESTINAL TRACT USE]
a82..	DIPHENOXYLATE HYDROCHLORIDE
a83..	LOPERAMIDE HYDROCHLORIDE
a84..	OPIUM AND MORPHINE
a85..	LOPERAMIDE HYDROCHLORIDE+SIMETHICONE
a9...	OTHER ANTIDIARRHOEAL PREPARATIONS
a91..	OTHER ANTIDIARRHOEAL PREPARATIONS A-Z
aa...	CHRONIC DIARRHOEA TREATMENT
aa1..	AZATHIOPRINE [GASTROINTESTINAL]
aa2..	COLESTYRAMINE [ANTIDIARRHOEAL USE]
aa3..	MESALAZINE
aa4..	PREDNISOLONE [for preparations see rectal prednisolone]
aa5..	SODIUM CROMOGLICATE [GASTRO-INTESTINAL USE]
aa6..	SULFASALAZINE [GASTRO-INTESTINAL USE]
aa7..	OLSALAZINE SODIUM
aa8..	*MESALAZINE DUPLICATE DELETED
aa9..	BUDESONIDE [GASTRO-INTESTINAL USE]
aaA..	BALSALAZIDE DISODIUM
aaB..	BECLOMETASONE [GASTRO-INTESTINAL USE]
ab...	BULK FORMING DRUGS [GASTRO-INTESTINAL USE]
ab1..	BRAN
ab2..	ISPAGHULA HUSK [GASTRO-INTESTINAL BULK LAXATIVE]
ab3..	METHYLCELLULOSE [GASTRO-INTESTINAL BULK LAXATIVE]
ab4..	STERCULIA [BULK LAXATIVE]
ac...	STIMULANT LAXATIVES
ac1..	BISACODYL
ac2..	CASCARA
ac3..	CASTOR OIL [LAXATIVE]

ac4..	DANTRON
ac5..	DOCUSATE SODIUM
ac6..	FIG
ac7..	SENNA
ac8..	SODIUM PICOSULFATE
ad...	FAECAL SOFTENERS
ad1..	LIQUID PARAFFIN [LAXATIVE]
ae...	OSMOTIC LAXATIVES
ae1..	LACTULOSE
ae2..	MAGNESIUM HYDROXIDE [LAXATIVE]
ae3..	MAGNESIUM SULFATE
ae4..	POLYETHYLENE GLYCOL ELECTROLYTE
ae5..	MAGNESIUM CITRATE
ae6..	LACTITOL
ae7..	SODIUM PHOSPHATE
af...	RECTALLY GIVEN LAXATIVES
af1..	RECTAL LAXATIVES A-Z
ag...	OTHER LAXATIVES
ag1..	OTHER LAXATIVES A-Z
ah...	RECTAL SOOTHING AGENTS
ah1..	RECTAL SOOTHER A-Z
ai...	RECTAL CORTICOSTEROIDS
ai1..	RECTAL HYDROCORTISONE
ai2..	RECTAL PREDNISOLONE
ai3..	RECTAL BUDESONIDE
aj...	RECTAL COMPOUNDS WITH STEROIDS
aj1..	RECTAL COMPOUNDS+STEROIDS A-Z
ak...	OTHER RECTAL PREPARATIONS
ak1..	PHENOL [RECTAL]
ak2..	RECTAL GLYCERYL TRINITRATE
al...	*STOMA CARE [see chapter s]
al1..	STOMA ADHESIVES [see chapter s]
al2..	STOMA ADHESIVE REMOVERS [see chapter s]
al3..	*STOMA DEODORANTS [see chap s]
al4..	STOMA SKIN "HELPERS" [see chapter s]
al5..	*STOMA PATIENT PRESCRIBING
am...	GALL BLADDER DRUGS
am1..	CHENODEOXYCHOLIC ACID
am2..	DEHYDROCHOLIC ACID
am3..	URSODEOXYCHOLIC ACID
am4..	OTHER BILIARY DRUGS
am5..	CHENO- + URSO- DEOXYCHOLIC ACID
an...	GASTRIC ACIDITY INCREASE DRUGS
an1..	GASTRIC ACIDITY INCREASERS A-Z
ao...	APROTININ [PANCREATITIS] [see chapter b]

ao1..	APROTININ [PANCREATITIS] [see chapter b]
ap...	PANCREATIN SUPPLEMENTS
ap1..	PANCREATIN
aq...	EMETICS
aq1..	IPECACUANHA
ar...	ADSORBENTS
ar1..	CHARCOAL ACTIVATED
ar2..	FULLER'S EARTH
as...	CYANIDE POISONING [ANTIDOTE]
as1..	DICOBALT EDETATE
as2..	SODIUM NITRITE
as3..	SODIUM THIOSULFATE
as4..	AMYL NITRITE
at...	HEAVY METAL POISONING [ANTIDOTE]
at1..	DIMERCAPROL
at2..	SODIUM CALCIUM EDETATE
au...	ORGANOPHOSPHORUS POISONING
au1..	PRALIDOXIME MESYLATE
au2..	PRALIDOXIME CHLORIDE
av...	HELICOBACTER PYLORI DIAGNOSTIC TEST
av1..	UREA [13C]
aw...	PERIPHERALLY ACTING ANTIOBESITY DRUGS
aw1..	ORLISTAT
ax...	OPIOID ANTAGONIST
ax1..	METHYLNALTREXONE BROMIDE
ax2..	NALOXEGOL
ay...	ANTIDIARRHOEAL ANTISECRETORY
ay1..	RACECADOTRIL
az...	OTHER GASTROINTESTINAL DRUGS
az1..	TEDUGLUTIDE
b....	CARDIOVASCULAR DRUGS
b1...	CARDIAC GLYCOSIDES
b11..	DIGOXIN
b12..	DIGITOXIN
b13..	*LANATOSIDE C
b14..	*MEDIGOXIN
b15..	*OUABAIN
b16..	DIGOXIN SPECIFIC ANTIBODY
b2...	THIAZIDE DIURETICS
b21..	BENDROFLUMETHIAZIDE
b22..	CHLOROTHIAZIDE
b23..	CHLORTALIDONE
b24..	CLOPAMIDE [INGREDIENT see bdek]
b25..	CYCLOPENTHIAZIDE
b26..	HYDROCHLOROTHIAZIDE

b27..	HYDROFLUMETHIAZIDE
b28..	INDAPAMIDE
b29..	MEFRUSIDE
b2a..	*METHYCLOTHIAZIDE
b2b..	METOLAZONE
b2c..	POLYTHIAZIDE
b2d..	XIPAMIDE
b3...	LOOP DIURETICS
b31..	FUROSEMIDE
b32..	BUMETANIDE
b33..	ETACRYNIC ACID
b34..	PIRETANIDE
b35..	TORASEMIDE
b4...	POTASSIUM SPARING DIURETICS
b41..	AMILORIDE HYDROCHLORIDE
b42..	POTASSIUM CANRENOATE
b43..	SPIRONOLACTONE
b44..	TRIAMTERENE
b45..	EPLERENONE
b5...	POTASSIUM SPARING COMPOUND DIURETICS
b51..	POTASSIUM SPARING COMPOUND DIURETICS A-Z
b6...	OSMOTIC DIURETICS
b61..	MANNITOL
b62..	*UREA
b7...	MERCURIAL DIURETICS
b71..	MERSALYL
b8...	CARBONIC ANHYDRASE INHIBITORS [NO DRUGS HERE]
b9...	DIURETICS+POTASSIUM SUPPLEMENT
b91..	DIURETICS+POTASSIUM SUPPLEMENT A-Z
ba...	INDIVIDUAL ARRHYTHMIAS [NO DRUGS HERE]
bA...	CALCIUM-CHANNEL BLOCKER+ANGIOTENSIN-CONVERTING ENZYME INHIBITOR
bA1..	FELODIPINE+RAMIPRIL
bb...	SUPRAVENTRICULAR ARRHYTHMIAS
bB...	IF INHIBITOR
bb1..	AMIODARONE HYDROCHLORIDE
bB1..	IVABRADINE
bb2..	ATROPINE SULFATE [ANTIARRHYTHMIC]
bb3..	VERAPAMIL HYDROCHLORIDE
bb4..	DRONEDARONE HYDROCHLORIDE
bc...	VENTRICULAR ARRHYTHMIA DRUGS
bC...	BRADYKININ ANTAGONIST
bc1..	BRETYLIUM TOSYLATE
bC1..	ICATIBANT
bc2..	DISOPYRAMIDE
bc3..	FLECAINIDE ACETATE

bc4..	LIDOCAINE HYDROCHLORIDE [ANTIARRHYTHMIC]
bc5..	MEXILETINE HYDROCHLORIDE
bc6..	PHENYTOIN SODIUM [ANTIARRHYTHMIC]
bc7..	*PRACTOLOL [ANTIARRHYTHMIC]
bc8..	PROCAINAMIDE HYDROCHLORIDE
bc9..	QUINIDINE
bca..	TOCAINIDE HYDROCHLORIDE
bcb..	PROPAFENONE HYDROCHLORIDE
bcc..	ADENOSINE
bcd..	MORACIZINE HYDROCHLORIDE
bd...	BETA-ADRENOCEPTOR BLOCKERS
bD...	OTHER ANTIANGINAL DRUGS
bd1..	PROPRANOLOL HYDROCHLORIDE
bD1..	RANOLAZINE
bd2..	ACEBUTOLOL
bd3..	ATENOLOL
bd4..	BETAXOLOL HCL [B-BLOCKER]
bd5..	LABETALOL HYDROCHLORIDE
bd6..	METOPROLOL TARTRATE
bd7..	NADOLOL
bd8..	OXPRENOLOL HYDROCHLORIDE
bd9..	PENBUTOLOL SULPHATE[INGREDIENT]
bda..	PINDOLOL
bdb..	*PRACTOLOL [B-BLOCKER]
bdc..	SOTALOL HYDROCHLORIDE
bdd..	TIMOLOL MALEATE [B-BLOCKER]
bde..	COMPOUND BETA-BLOCKERS
bdf..	BISOPROLOL FUMARATE
bdg..	*XAMOTEROL FUMARATE
bdh..	*METOPROLOL FUMARATE
bdi..	*CARTEOLOL HYDROCHLORIDE
bdj..	CELIPROLOL HYDROCHLORIDE
bdk..	ESMOLOL HYDROCHLORIDE
bdl..	CARVEDILOL
bdm..	NEBIVOLOL
bdn..	PROPRANOLOL HYDROCHLORIDE [2]
be...	VASODILATOR ANTIHYPERTENSIVES
bE...	OTHER CARDIAC PREPARATIONS
be1..	DIAZOXIDE [CARDIOVASCULAR USE]
bE1..	REGADENOSON
be2..	HYDRALAZINE HYDROCHLORIDE
be3..	MINOXIDIL
be4..	SODIUM NITROPRUSSIDE
bf...	CENTRAL ANTIHYPERTENSIVES
bf1..	CLONIDINE HYDROCHLORIDE [ANTIHYPERTENSIVE]

Wilkinson T, *et al. J Epidemiol Community Health* 2022; 76:223–229. doi: 10.1136/jech-2021-217090

bf2..	METHYLDOPA
bf3..	RESERPINE/RAUWOLFIA ALKALOIDS
bf4..	MOXONIDINE
bg...	ADRENERGIC NEURONE BLOCKERS
bg1..	BETANIDINE SULFATE
bg2..	DEBRISOQUINE
bg3..	GUANETHIDINE MONOSULFATE [ANTIHYPERTENSIVE]
bh...	ALPHA-ADRENOCEPTOR BLOCKERS
bh1..	INDORAMIN
bh2..	PHENOXYBENZAMINE HYDROCHLORIDE [CARDIOVASCULAR USE]
bh3..	PHENTOLAMINE MESYLATE
bh4..	PRAZOSIN HYDROCHLORIDE
bh5..	TERAZOSIN HYDROCHLORIDE
bh6..	DOXAZOSIN
bi...	ANGIOTENSIN-CONVERTING ENZYME INHIBITORS
bi1..	CAPTOPRIL
bi2..	ENALAPRIL MALEATE
bi3..	LISINOPRIL
bi4..	QUINAPRIL
bi5..	PERINDOPRIL ERBUMINE
bi6..	RAMIPRIL
bi7..	SODIUM FOSINOPRIL
bi8..	CILAZAPRIL
bi9..	TRANDOLAPRIL
biA..	MOEXIPRIL
biB..	IMIDAPRIL HYDROCHLORIDE
biC..	PERINDOPRIL ARGININE
bj...	GANGLION BLOCKING DRUGS
bj1..	TRIMETAPHAN
bk...	OTHER ANTIHYPERTENSIVES
bk1..	METIROSINE
bk2..	ANTIHYPERTENSIVES+BARBITURATES
bk3..	LOSARTAN
bk4..	VALSARTAN
bk5..	IRBESARTAN
bk6..	TRANDOLAPRIL+VERAPAMIL HYDROCHLORIDE
bk7..	CANDESARTAN CILEXETIL
bk8..	TELMISARTAN
bk9..	EPROSARTAN
bkA..	BOSENTAN
bkB..	OLMESARTAN
bkC..	HYDROCHLOROTHIAZIDE + OLMESARTAN
bkD..	AMLODIPINE + VALSARTAN
bkE..	SITAXENTAN
bkF..	ALISKIREN

bkG..	AMBRISENTAN
bkH..	OLMESARTAN+AMLODIPINE
bkI..	OLMESARTAN+AMLODIPINE+HYDROCHLOROTHIAZIDE
bkJ..	AZILSARTAN
bkK..	MACITENTAN
bl...	VASODILATORS USED IN ANGINA PECTORIS
bl1..	GLYCERYL TRINITRATE
bl2..	ISOSORBIDE DINITRATE
bl3..	ISOSORBIDE MONONITRATE
bl4..	PENTAERITHRITYL TETRANITRATE
bl5..	DILTIAZEM HYDROCHLORIDE
bl6..	*LIDOFLAZINE
bl7..	NICARDIPINE HYDROCHLORIDE
bl8..	NIFEDIPINE
bl9..	*PRENYLAMINE
bla..	ISRADIPINE
blb..	AMLODIPINE
blc..	FELODIPINE
bld..	*FLOSEQUINAN
ble..	LACIDIPINE
blf..	NICORANDIL
blg..	NISOLDIPINE
blh..	LERCANIDIPINE HYDROCHLORIDE
bli..	MIBEFRADIL
blj..	DILTIAZEM HYDROCHLORIDE 2
blk..	ISOSORBIDE MONONITRATE 2
bll..	NIFEDIPINE [2]
blm..	ISOSORBIDE MONONITRATE+ASPIRIN
bly..	ISOSORBIDE DINITRATE [GENERIC ADDITIONS]
blz..	GLYCERYL TRINITRATE [GENERIC ADDITIONS]
bm...	VASODILATORS IN HEART FAILURE [NO DRUGS HERE]
bn...	PERIPHERAL VASODILATORS
bn1..	*BAMETHAN SULPHATE
bn2..	CINNARIZINE [VASODILATOR]
bn3..	NICOTINIC ACID DERIVATIVES
bn4..	PENTOXIFYLLINE
bn5..	MOXISYLYTE
bn6..	OTHER PERIPHERAL VASCULAR DISEASE DRUGS
bn7..	ILOPROST
bo...	CEREBRAL VASODILATORS
bo1..	CO-DERGOCRINE MESYLATE
bo2..	*CYCLANDELATE
bo3..	ISOXSUPRINE HYDROCHLORIDE [CARDIOVASCULAR USE]
bo4..	NAFTIDROFURYL OXALATE
bp...	ADRENALINE + INOTROPICS

bp1..	ADRENALINE [CARDIOVASCULAR USE]
bp2..	DOBUTAMINE HYDROCHLORIDE
bp3..	DOPAMINE HYDROCHLORIDE
bp4..	ISOPRENALINE HYDROCHLORIDE
bp5..	XAMOTEROL [SYMPATHOMIMETIC]
bp6..	DOPEXAMINE HYDROCHLORIDE
bq...	VASOCONSTRICTING SYMPATHOMIMETIC
bq1..	METARAMINOL
bq2..	METHOXAMINE HYDROCHLORIDE
bq3..	NORADRENALINE ACID TARTRATE
bq4..	*OXEDRINE TARTRATE
bq5..	PHENYLEPHRINE HYDROCHLORIDE [CVS SYMPATHOMIMETIC]
bq6..	EPHEDRINE HYDROCHLORIDE
bq7..	MIDODRINE
br...	PARENTERAL ANTICOAGULANTS
br1..	HEPARIN INTRAVENOUS
br2..	HEPARIN SUBCUTANEOUS
br3..	HEPARIN FLUSHES
br4..	ANCROD
br5..	EPOPROSTENOL
br6..	ENOXAPARIN
br7..	TINZAPARIN SODIUM
br8..	DANAPAROID SODIUM
br9..	CERTOPARIN SODIUM
brA..	LEPIRUDIN
brB..	DESIRUDIN
brC..	REVIPARIN SODIUM
brD..	FONDAPARINUX
brE..	BEMIPARIN
brF..	BIVALIRUDIN
brG..	ARGATROBAN
brH..	DEFIBROTIDE
bs...	ORAL ANTICOAGULANTS
bs1..	WARFARIN SODIUM
bs2..	ACENOCOUMAROL
bs3..	PHENINDIONE
bs4..	DABIGATRAN ETEXILATE
bs5..	*DICOUMAROL [NO DRUGS HERE]
bs6..	RIVAROXABAN
bs7..	APIXABAN
bs8..	EDOXABAN
bt...	PROTAMINE SULFATE
bt1..	PROTAMINE SULFATE
bu...	ANTIPLATELET DRUGS
bu1..	DIPYRIDAMOLE

Wilkinson T, *et al. J Epidemiol Community Health* 2022; 76:223–229. doi: 10.1136/jech-2021-217090

bu2..	ASPIRIN [ANTIPLATELET]
bu3..	ABCIXIMAB
bu4..	DIPYRIDAMOLE+ASPIRIN
bu5..	CLOPIDOGREL
bu6..	TICLOPIDINE
bu7..	EPTIFIBATIDE
bu8..	TIROFIBAN
bu9..	CILOSTAZOL
buA..	PRASUGREL
buB..	TICAGRELOR
bv...	FIBRINOLYTIC DRUGS
bv1..	STREPTOKINASE
bv2..	UROKINASE
bv3..	ALTEPLASE
bv4..	ANISTREPLASE
bv5..	RETEPLASE
bv6..	TENECTEPLASE
bv7..	DROTRECOGIN ALFA (ACTIVATED)
bw...	ANTIFIBRINOLYTICS/HAEMOSTATICS
bw1..	*ETAMSYLATE
bw2..	*THROMBOPLASTIN
bw3..	TRANEXAMIC ACID
bw5..	*APROTININ [FIBRINOLYTIC]
bw6..	FACTOR VIII
bw7..	COLLAGEN [HAEMOSTATIC]
bw8..	FACTOR IX
bw9..	FACTOR VIIa
bwA..	FACTOR VIII+VON WILLEBRAND FACTOR
bwB..	FACTOR XIII PRODUCTS
bwC..	FIBRINOGEN+THROMBIN
bwD..	ANTITHROMBIN III
bwE..	HUMAN PROTHROMBIN COMPLEX
bwF..	ROMIPLOSTIM
bwG..	ELTROMBOPAG OLAMINE
bwH..	HUMAN FIBRINOGEN
bwI..	VON WILLEBRAND FACTOR
bwJ..	FACTOR VIII (2)
bx...	LIPID-LOWERING DRUGS
bx1..	BEZAFIBRATE
bx2..	COLESTYRAMINE [LIPID LOWERING USE]
bx3..	CLOFIBRATE
bx4..	COLESTIPOL HYDROCHLORIDE
bx5..	*DEXTROTHYROXINE SODIUM
bx6..	GEMFIBROZIL
bx7..	*NICOFURANOSE

Wilkinson T, *et al. J Epidemiol Community Health* 2022; 76:223–229. doi: 10.1136/jech-2021-217090

bx8..	NICOTINIC ACID
bx9..	PROBUCOL
bxa..	OMEGA-3 MARINE TRIGLYCERIDES
bxb..	ACIPIMOX
bxc..	FENOFIBRATE
bxd..	SIMVASTATIN
bxe..	PRAVASTATIN SODIUM
bxf..	CIPROFIBRATE
bxg..	FLUVASTATIN SODIUM
bxh..	ISPAGHULA HUSK [LIPID LOWERING USE]
bxi..	ATORVASTATIN
bxj..	CERIVASTATIN
bxk..	ROSUVASTATIN
bxl..	EZETIMIBE
bxm..	COLESEVELAM HYDROCHLORIDE
bxn..	LOMITAPIDE
bxo..	EVOLOCUMAB
by...	LOCAL SCLEROSANTS
by1..	ETHANOLAMINE OLEATE
by2..	SODIUM TETRADECYL SULFATE
bz...	POSITIVE INOTROPIC DRUGS
bz1..	ENOXIMONE
bz2..	MILRINONE
c....	RESPIRATORY DRUGS
c1...	SELECTIVE BETA-ADRENOCEPTOR STIMULANT
c11..	SALBUTAMOL [ORAL PREPARATIONS]
c12..	SALBUTAMOL [PARENTERAL PREPARATIONS]
c13..	SALBUTAMOL [INHALATION PREPRATIONS]
c14..	TERBUTALINE SULFATE [RESPIRATORY USE]
c15..	FENOTEROL HYDROBROMIDE
c16..	PIRBUTEROL
c17..	REPROTEROL HYDROCHLORIDE
c18..	RIMITEROL HYDROBROMIDE
c19..	SALMETEROL XINAFOATE
c1a..	TULOBUTEROL HYDROCHLORIDE
c1A..	SALINE FOR NEBULISATION
c1b..	INDACATEROL
c1B..	BAMBUTEROL HYDROCHLORIDE
c1c..	FLUTICASONE PROPIONATE+FORMOTEROL FUMARATE
c1C..	FORMOTEROL
c1d..	OLODATEROL
c1D..	SALMETEROL+FLUTICASONE PROPIONATE
c1e..	INDACATEROL+GLYCOPYRRONIUM
c1E..	SALBUTAMOL [INHALATION PREPRATIONS 2]
c2...	OTHER ADRENOCEPTOR STIMULANTS

c21..	ADRENALINE [RESP]
c22..	EPHEDRINE HYDROCHLORIDE [RESPIRATORY USE]
c23..	*ISOETHARINE HYDROCHLORIDE
c24..	ISOPRENALINE SULFATE
c25..	ORCIPRENALINE SULFATE [RESPIRATORY USE]
c3...	ANTICHOLINERGIC BRONCHODILATORS
c31..	IPRATROPIUM BROMIDE [1]
c32..	OXITROPIUM BROMIDE
c33..	TIOTROPIUM
c34..	ACLIDINIUM
c35..	UMECLIDINIUM
c4...	XANTHINE BRONCHODILATORS
c41..	AMINOPHYLLINE
c42..	CHOLINE THEOPHYLLINATE
c43..	THEOPHYLLINE
c44..	CAFFEINE
c5...	COMPOUND BRONCHODILATORS
c51..	COMPOUND BRONCHODILATORS A-Z
c52..	*BRONCHODILATORS + SEDATIVE
c53..	COMPOUND BRONCHODILATORS [1]
c6...	CORTICOSTEROIDS [RESPIRATORY USE]
c61..	BECLOMETASONE DIPROPIONATE [RESPIRATORY USE]
c62..	BECLOMETASONE COMPOUNDS
c63..	*BETAMETHASONE VALERATE
c64..	BUDESONIDE [RESPIRATORY USE]
c65..	FLUTICASONE PROPIONATE [RESPIRATORY USE]
c66..	BECLOMETASONE DIPROPIONATE [RESPIRATORY USE 2]
c67..	BUDESONIDE+FORMOTEROL
c68..	MOMETASONE [RESPIRATORY USE]
c69..	CICLESONIDE
c6A..	BECLOMETASONE+FORMOTEROL
c6B..	FLUTICASONE+VILANTEROL
c7...	ASTHMA PROPHYLAXIS
c71..	SODIUM CROMOGLICATE [ASTHMA]
c72..	SODIUM CROMOGLICATE COMPOUNDS
c73..	KETOTIFEN [ASTHMA PROPHYLAXIS]
c74..	NEDOCROMIL SODIUM [ASTHMA]
c8...	ANTIHISTAMINES
c81..	ASTEMIZOLE
c82..	AZATADINE MALEATE
c83..	BROMPHENIRAMINE MALEATE
c84..	CHLORPHENAMINE MALEATE
c85..	CLEMASTINE
c86..	CYPROHEPTADINE HYDROCHLORIDE
c87..	DIMETHINDENE MALEATE

c88..	DIPHENHYDRAMINE HYDROCHLORIDE [ANTIHISTAMINE]
c89..	DIPHENYLPYRALINE HYDROCHLORIDE
c8a..	HYDROXYZINE HYDROCHLORIDE [ANTIHISTAMINE]
c8b..	KETOTIFEN [ANTIHISTAMINE]
c8c..	MEBHYDROLIN
c8d..	MEPYRAMINE MALEATE [ANTIHISTAMINE]
c8e..	MEQUITAZINE
c8f..	OXATOMIDE
c8g..	PHENINDAMINE TARTRATE
c8h..	PHENIRAMINE MALEATE
c8i..	PROMETHAZINE HYDROCHLORIDE [ANTIHISTAMINE]
c8j..	TERFENADINE
c8k..	ALIMEMAZINE TARTRATE [ANTIHISTAMINE]
c8l..	TRIPROLIDINE HYDROCHLORIDE
c8m..	ACRIVASTINE
c8n..	CETIRIZINE DIHYDROCHLORIDE
c8o..	LORATADINE
c8p..	FEXOFENADINE
c8q..	MIZOLASTINE
c8r..	DESLORATADINE
c8s..	LEVOCETIRIZINE
c8t..	EPINASTINE
c8u..	RUPATADINE
c8v..	BILASTINE
c9...	HYPOSENSITISATION
c91..	POLLEN ALLERGY PREPARATIONS
c92..	HOUSE DUST MITE ALLERGY PREPS.
c93..	*SPECIFIC ALLERGY PREPARATIONS
c94..	WASP/BEE VENOM ALLERGY PREPARATIONS
c95..	ALLERGY TESTING PREPARATIONS
ca...	ALLERGIC EMERGENCIES [NO DRUGS HERE]
cA...	LEUKOTRIENE RECEPTOR ANTAGONIST
cA1..	MONTELUKAST
cA2..	ZAFIRLUKAST
cb...	RESPIRATORY STIMULANTS + SURFACTANTS
cb1..	DOXAPRAM HYDROCHLORIDE [RESPIRATORY USE]
cb2..	NIKETHAMIDE
cb3..	ETHAMIVAN
cb4..	COLFOSCERIL PALMITATE
cb5..	BERACTANT
cb6..	PHOSPHOLIPID FRACTION
cb7..	PUMACTANT
cc...	OXYGEN
cd...	INHALATIONAL MUCOLYTICS
cd1..	*TYLOXAPOL

Wilkinson T, *et al. J Epidemiol Community Health* 2022; 76:223–229. doi: 10.1136/jech-2021-217090

cd2..	DORNASE ALFA
ce...	ORAL/PARENTERAL MUCOLYTICS
ce1..	ACETYLCYSTEINE [RESPIRATORY USE]
ce2..	*BROMHEXINE HYDROCHLORIDE
ce3..	CARBOCISTEINE
ce4..	MECYSTEINE HYDROCHLORIDE
ce5..	ERDOSTEINE
cf...	INHALATIONS
cf1..	INHALATIONS A-Z
cg...	COUGH SUPPRESSANTS
cg1..	CODEINE PHOSPHATE
cg2..	DEXTROMETHORPHAN HYDROBROMIDE
cg3..	DIAMORPHINE HCL [COUGH]
cg4..	ISOAMINILE CITRATE
cg5..	METHADONE HCL [COUGH]
cg6..	NOSCAPINE
cg7..	PHOLCODINE
ch...	EXPECTORANTS/COUGH COMPOUNDS
ch1..	GENERIC EXPECTORANTS/COUGH COMPOUND PREPARATIONS
ch2..	PROPRIETARY COUGH COMPOUND PREPARATIONS A-L
ch3..	PROPRIETARY COUGH COMPOUND PREPARATIONS M-Z
ci...	SYSTEMIC NASAL DECONGESTANTS
ci1..	EPHEDRINE SYSTEMIC DECONGESTANTS
cj...	AMINOGLYCOSIDES [RESPIRATORY USE]
cj1..	TOBRAMYCIN [RESPIRATORY USE]
ck...	MONOCLONAL IgE ANTIBODY
ck1..	OMALIZUMAB
cl...	PDE4 INHIBITORS
cl1..	ROFLUMILAST
cm...	CFTR SELECTIVE POTENTIATOR
cm1..	IVACAFTOR
d....	CENTRAL NERVOUS SYSTEM DRUGS
d1...	HYPNOTICS
d11..	CHLORAL HYDRATE
d12..	CLOMETHIAZOLE EDISYLATE [HYPNOTIC]
d13..	*DICHLORALPHENAZONE
d14..	*FLUNITRAZEPAM
d15..	FLURAZEPAM
d16..	LOPRAZOLAM
d17..	LORMETAZEPAM
d18..	NITRAZEPAM
d19..	PROMETHAZINE HCL [HYPNOTIC] see section c8i..
d1a..	TEMAZEPAM [HYPNOTIC]
d1b..	*TRIAZOLAM
d1c..	TRICLOFOS SODIUM

d1d..	ZOPICLONE
d1e..	*GLUTETHIMIDE [no drugs here]
d1f..	ZOLPIDEM
d1g..	ZALEPLON
d1h..	MELATONIN
d1i..	DEXMEDETOMIDINE
d2...	ANXIOLYTICS
d21..	DIAZEPAM [ANXIOLYTIC]
d22..	ALPRAZOLAM
d23..	BROMAZEPAM
d24..	CHLORDIAZEPOXIDE
d25..	CHLORMEZANONE
d26..	CLOBAZAM
d27..	CLORAZEPATE DIPOTASSIUM
d28..	HYDROXYZINE HCL [ANXIOLYTIC]
d29..	*KETAZOLAM
d2a..	LORAZEPAM [ANXIOLYTIC]
d2b..	*MEDAZEPAM
d2c..	MEPROBAMATE
d2d..	OXAZEPAM
d2e..	*PRAZEPAM
d2f..	BUSPIRONE HYDROCHLORIDE
d2g..	FLUMAZENIL
d3...	BARBITURATES/METHYPRYLONE
d31..	BARBITURATES
d32..	*METHYPRYLONE
d4...	ANTIPSYCHOTIC DRUGS
d41..	CHLORPROMAZINE HYDROCHLORIDE
d42..	BENPERIDOL
d43..	*CHLORPROTHIXENE
d44..	DROPERIDOL [CENTRAL NERVOUS SYSTEM USE]
d45..	FLUPENTIXOL [ANTIPSYCHOTIC]
d46..	FLUPHENAZINE HYDROCHLORIDE
d47..	HALOPERIDOL [ANTIPSYCHOTIC]
d48..	LEVOMEPROMAZINE
d49..	OXYPERTINE
d4a..	PERICYAZINE
d4b..	PERPHENAZINE [CENTRAL NERVOUS SYSTEM USE]
d4c..	PIMOZIDE
d4d..	PROCHLORPERAZINE [antipsych] [see dhe..]
d4e..	PROMAZINE HYDROCHLORIDE
d4f..	SULPIRIDE
d4g..	THIORIDAZINE
d4h..	TRIFLUOPERAZINE [ANTIPSYCHOTIC]
d4i..	TRIFLUPERIDOL

d4j..	ZUCLOPENTHIXOL DIHYDROCHLORIDE
d4k..	LOXAPINE SUCCINATE
d4l..	CLOZAPINE
d4m..	REMOXIPRIDE
d4n..	ZUCLOPENTHIXOL ACETATE
d4p..	RISPERIDONE
d4q..	SERTINDOLE
d4r..	OLANZAPINE
d4s..	QUETIAPINE
d4t..	AMISULPRIDE
d4u..	ZOTEPINE
d4v..	ARIPIPRAZOLE
d4w..	PALIPERIDONE
d4x..	ASENAPINE
d4y..	LURASIDONE
d5...	ANTIPSYCHOTIC DEPOT INJECTIONS
d51..	FLUPENTIXOL DECANOATE
d52..	FLUPHENAZINE DECANOATE
d53..	*FLUPHENAZINE ENANTHATE
d54..	FLUSPIRILENE
d55..	HALOPERIDOL DECANOATE
d56..	PIPOTIAZINE PALMITATE
d57..	ZUCLOPENTHIXOL DECANOATE
d58..	OLANZAPINE PAMOATE
d6...	LITHIUM SALTS
d61..	LITHIUM CARBONATE
d62..	LITHIUM CITRATE
d7...	TRICYCLIC ANTIDEPRESSANTS
d71..	AMITRIPTYLINE HYDROCHLORIDE [ANTIDEPRESSANT]
d72..	*BUTRIPTYLINE
d73..	CLOMIPRAMINE HYDROCHLORIDE
d74..	DESIPRAMINE HYDROCHLORIDE
d75..	DOSULEPIN HYDROCHLORIDE
d76..	DOXEPIN
d77..	IMIPRAMINE HYDROCHLORIDE [ANTIDEPRESSANT]
d78..	IPRINDOLE
d79..	LOFEPRAMINE
d7a..	MAPROTILINE HYDROCHLORIDE
d7b..	MIANSERIN HYDROCHLORIDE
d7c..	NORTRIPTYLINE
d7d..	PROTRIPTYLINE HYDROCHLORIDE
d7e..	TRAZODONE HYDROCHLORIDE
d7f..	TRIMIPRAMINE
d7g..	VILOXAZINE HYDROCHLORIDE
d7h..	AMOXAPINE

Wilkinson T, *et al. J Epidemiol Community Health* 2022; 76:223–229. doi: 10.1136/jech-2021-217090

d8...	MONOAMINE-OXIDASE INHIBITORS
d81..	PHENELZINE
d82..	*IPRONIAZID
d83..	ISOCARBOXAZID
d84..	TRANYLCYPROMINE
d85..	MOCLOBEMIDE
d9...	COMPOUND ANTIDEPRESSANT DRUGS
d91..	COMPOUND ANTIDEPRESSANTS A-Z
da...	OTHER ANTIDEPRESSANT DRUGS
dA...	OTHER DRUGS USED FOR THE TREATMENT OF MULTIPLE SCLEROSIS
da1..	FLUPENTIXOL [ANTIDEPRESSANT]
dA1..	GLATIRAMER
da2..	TRYPTOPHAN
dA2..	CANNABINOIDS
da3..	FLUVOXAMINE MALEATE
dA3..	FAMPRIDINE
da4..	FLUOXETINE HYDROCHLORIDE
dA4..	TERIFLUNOMIDE
da5..	SERTRALINE HYDROCHLORIDE
dA5..	DIMETHYL FUMARATE
da6..	PAROXETINE HYDROCHLORIDE
da7..	VENLAFAXINE
da8..	NEFAZODONE
da9..	CITALOPRAM
daA..	REBOXETINE
daB..	MIRTAZAPINE
daC..	ESCITALOPRAM
daD..	AGOMELATINE
daE..	VORTIOXETINE
db...	WEAK CENTRAL NERVOUS SYSTEM STIMULANTS
dB...	OTHER ANTIDEMENTIA DRUGS
db1..	*PEMOLINE
dB1..	MEMANTINE HYDROCHLORIDE
db2..	WEAK CENTRAL NERVOUS SYSTEM STIMULANT + VITAMINS
dc...	AMFETAMINE AND COCAINE
dC...	OTHER DRUGS USED FOR THE TREATMENT OF NARCOLEPSY
dc1..	DEXAMFETAMINE SULFATE
dC1..	SODIUM OXYBATE
dc2..	*COCAINE [NO DRUGS HERE]
dD...	OTHER CENTRAL NERVOUS SYSTEM DRUGS
dD1..	TAFAMIDIS
de...	BULK-FORMING DRUGS [APPETITE SUPPRESSANTS]
de1..	METHYLCELLULOSE [APPETITE SUPPRESSANTS]
de2..	STERCULIA [APPETITE SUPPRESSANT]
df...	CENTRAL APPETITE SUPPRESSANTS

df1..	DIETHYLPROPION HYDROCHLORIDE
df2..	FENFLURAMINE HYDROCHLORIDE
df3..	MAZINDOL
df4..	PHENTERMINE
df5..	DEXFENFLURAMINE
df6..	SIBUTRAMINE
df7..	RIMONABANT
dh...	NAUSEA AND VERTIGO DRUGS
dh1..	BETAHISTINE HYDROCHLORIDE
dh2..	CHLORPROMAZINE HCL [NAUSEA] [see dh2..]
dh3..	CINNARIZINE [NAUSEA]
dh4..	CYCLIZINE
dh5..	DIMENHYDRINATE
dh6..	DIPHENHYDRAMINE HYDROCHLORIDE [NAUSEA]
dh7..	DOMPERIDONE (NAUSEA)
dh8..	HYOSCINE HYDROBROMIDE [CENTRAL NERVOUS SYSTEM USE]
dh9..	MECLOZINE HYDROCHLORIDE
dha..	*MEPYRAMINE MALEATE [NAUSEA]
dhb..	METOCLOPRAMIDE HYDROCHLORIDE
dhc..	NABILONE
dhd..	*PERPHENAZINE [NO DRUGS HERE]
dhe..	PROCHLORPERAZINE [NAUSEA]
dhf..	PROMETHAZINE [nausea] [see c8i..]
dhg..	PROMETHAZINE THEOCLATE
dhh..	*THIETHYLPERAZINE
dhi..	TRIFLUOPERAZINE [NAUSEA] [see dh4..]
dhj..	*CISAPRIDE
dhk..	ONDANSETRON
dhl..	GRANISETRON HYDROCHLORIDE
dhm..	TROPISETRON HYDROCHLORIDE
dho..	DOLASETRON
dhp..	APREPITANT
dhq..	PALONOSETRON
dhr..	CINNARIZINE+DIMENHYDRINATE
dhs..	FOSAPREPITANT
dht..	NETUPITANT+PALONOSETRON
di...	NON-NARCOTIC ANALGESICS
di1..	ASPIRIN [CENTRAL NERVOUS SYSTEM USE]
di2..	PARACETAMOL
di3..	BENORILATE [CENTRAL NERVOUS SYSTEM]
di4..	DIFLUNISAL [CNS analgesic] [seej23..]
di5..	*FENOPROFEN [ANALGESIC]
di6..	IBUPROFEN [CNS analgesic] [see j28..]
di7..	MEFENAMIC ACID [CNS ANALGESIC] [see j2b..]
di8..	NAPROXEN SODIUM

di9..	NEFOPAM HYDROCHLORIDE
dia..	COMPOUND ANALGESICS A-L
dib..	COMPOUND ANALGESICS M-Z
dic..	COMPOUND ANALGESICS[1]
did..	PARACETAMOL PRODUCTS[2]
die..	ZICONOTIDE
dj...	NARCOTIC ANALGESICS
dj1..	MORPHINE SALTS(1) [CENTRAL NERVOUS SYSTEM USE]
dj2..	MORPHINE ANALGESIC ELIXIRS
dj3..	BUPRENORPHINE
dj4..	CODEINE PHOSPHATE [ANALGESIC]
dj5..	DEXTROMORAMIDE
dj6..	DEXTROPROPOXYPHENE HYDROCHLORIDE
dj7..	DIAMORPHINE HCL [ANALGESIC]
dj8..	DIHYDROCODEINE TARTRATE
dj9..	DIPIPANONE HYDROCHLORIDE
dja..	LEVORPHANOL TARTRATE [CENTRAL NERVOUS SYSTEM USE]
djA..	TRAMADOL (2)
djb..	MEPTAZINOL [ANALGESIC]
djB..	TAPENTADOL
djc..	METHADONE HCL [ANALGESIC]
djd..	NALBUPHINE HYDROCHLORIDE [CENTRAL NERVOUS SYSTEM USE]
dje..	*PAPAVERETUM [CNS NARCOTIC]
djf..	PENTAZOCINE
djg..	PETHIDINE HYDROCHLOR [ANALGESIC]
djh..	PHENAZOCINE HYDROBROMIDE
dji..	TRAMADOL
djj..	HYDROMORPHONE HYDROCHLORIDE
djk..	OXYCODONE HYDROCHLORIDE
djl..	LEVACETYLMETHADOL HYDROCHLORIDE
djm..	OXYCODONE HYDROCHLORIDE (2)
djy..	MORPHINE SALTS(3) [CENTRAL NERVOUS SYSTEM USE]
djz..	MORPHINE SALTS(2) [CENTRAL NERVOUS SYSTEM USE]
dk...	TRIGEMINAL NEURALGIA [no drugs here]
dl...	ACUTE MIGRAINE TREATMENT
dl1..	ANALGESICS WITH ANTI-EMETICS
dl2..	ERGOTAMINE TARTRATE
dl3..	*DIHYDROERGOTAMINE MESYLATE
dl4..	ISOMETHEPTENE MUCATE
dl5..	SUMATRIPTAN SUCCINATE
dl6..	TOLFENAMIC ACID
dl7..	SUMATRIPTAN
dl8..	ZOLMITRIPTAN
dl9..	NARATRIPTAN
dlA..	RIZATRIPTAN

dlB..	DIHYDROERGOTAMINE
dlC..	ALMOTRIPTAN
dlD..	ELETRIPTAN
dlE..	FROVATRIPTAN
dm...	PROPHYLAXIS OF MIGRAINE
dm1..	CLONIDINE HCL [MIGRAINE]
dm2..	METHYSERGIDE
dm3..	PIZOTIFEN
dn...	CONTROL OF EPILEPSY
dn1..	ACETAZOLAMIDE [EPILEPSY]
dn2..	*BECLAMIDE
dn3..	CARBAMAZEPINE
dn4..	CLONAZEPAM [EPILEPSY CONTROL]
dn5..	ETHOSUXIMIDE
dn6..	METHYLPHENOBARBITAL
dn7..	PHENOBARBITAL
dn8..	PHENYTOIN
dn9..	PHENYTOIN SODIUM
dna..	PRIMIDONE
dnb..	SODIUM VALPROATE
dnc..	CLOBAZAM [EPILEPSY ONLY]
dne..	VIGABATRIN
dnf..	LAMOTRIGINE
dng..	PIRACETAM
dnh..	VALPROIC ACID
dni..	FOSPHENYTOIN SODIUM
dnj..	GABAPENTIN
dnk..	TOPIRAMATE
dnl..	TIAGABINE
dnm..	OXCARBAZEPINE
dno..	LEVETIRACETAM
dnp..	PREGABALIN
dnq..	ZONISAMIDE
dnr..	RUFINAMIDE
dns..	STIRIPENTOL
dnt..	LACOSAMIDE
dnu..	ESLICARBAZEPINE
dnv..	RETIGABINE
dnw..	PERAMPANEL
do...	STATUS EPILEPTICUS DRUGS
do1..	DIAZEPAM [EPILEPSY USE]
do2..	CLONAZEPAM [STATUS EPILEPSY]
do3..	CLOMETHIAZOLE EDISYLATE [CENTRAL NERVOUS SYSTEM USE]
do4..	LORAZEPAM [EPILEPSY]
do5..	PARALDEHYDE

do6..	PHENYTOIN SODIUM [STATUS EPILEPSY]
dp...	FEBRILE CONVULSIONS
dq...	PARKINSONISM-DOPAMINERGIC DRUG
dq1..	LEVODOPA
dq2..	LEVODOPA WITH BENSERAZIDE
dq3..	LEVODOPA WITH CARBIDOPA
dq4..	AMANTADINE HYDROCHLORIDE [PARKINSONS]
dq5..	BROMOCRIPTINE [PARKINSONS]
dq6..	SELEGILINE
dq7..	LISURIDE MALEATE
dq8..	PERGOLIDE MESYLATE
dq9..	APOMORPHINE HYDROCHLORIDE
dqA..	ROPINIROLE HYDROCHLORIDE
dqB..	CABERGOLINE [PARKINSONS]
dqC..	TOLCAPONE
dqD..	ENTACAPONE
dqE..	PRAMIPEXOLE
dqF..	RASAGILINE
dqG..	ROTIGOTINE
dr...	PARKINSONISM-ANTICHOLINERGICS
dr1..	TRIHEXYPHENIDYL HYDROCHLORIDE
dr2..	ORPHENADRINE HYDROCHLORIDE
dr3..	BENZATROPINE MESYLATE
dr4..	BIPERIDEN
dr5..	METHIXENE HYDROCHLORIDE
dr6..	PROCYCLIDINE HYDROCHLORIDE
ds...	ESSENTIAL TREMOR,CHOREA,TICS
ds1..	HALOPERIDOL [TICS, CHOREA] [see d47..]
ds2..	TETRABENAZINE
ds3..	CLOSTRIDIUM BOTULINUM A TOXIN-HAEMAGGLUTININ COMPLEX
ds4..	CLOSTRIDIUM BOTULINUM B TOXIN
dt...	CEREBRAL VESSEL SPASMOLYTIC
dt1..	NIMODIPINE
du...	ALCOHOL/DRUG DEPENDENCY DRUGS
du1..	DISULFIRAM
du2..	NALTREXONE HYDROCHLORIDE
du3..	NICOTINE
du4..	LOFEXIDINE HYDROCHLORIDE
du5..	ACAMPROSATE CALCIUM
du6..	BUPROPION
du7..	NICOTINE 2
du8..	VARENICLINE
du9..	NICOTINE WITHDRAWAL PRODUCTS
duA..	NALMEFENE
duB..	NICOTINE 3

dv...	PARACETAMOL POISONING
dv1..	ACETYLCYSTEINE [PARACETAMOL POISONING TREATMENT]
dv2..	METHIONINE
dw...	DRUGS USED TO TREAT HYPERACTIVITY DISORDERS
dw1..	METHYLPHENIDATE
dw2..	ATOMOXETINE
dw3..	LISDEXAMFETAMINE
dx...	DRUGS USED FOR THE TREATMENT OF MOTOR NEURONE DISEASE
dx1..	RILUZOLE
dy...	CENTRAL ACETYLCHOLINESTERASE INHIBITOR
dy1..	DONEPEZIL HYDROCHLORIDE
dy2..	RIVASTIGMINE
dy3..	GALANTAMINE
dz...	OTHER CENTRAL NERVOUS SYSTEM STIMULANTS
dz1..	MODAFINIL
e....	DRUGS USED IN INFECTIONS
e1...	PENICILLINASE SENS PENICILLINS
e11..	BENZYLPENICILLIN(PENICILLIN G)
e12..	*BENETHAMINE PENICILLIN
e13..	*BENZATHINE PENICILLIN
e14..	*PHENETHICILLIN
e15..	PHENOXYMETHYLPENICILLIN
e16..	PROCAINE PENICILLIN
e2...	PENICILLINASE-RESISTANT PENICILLINS
e21..	CLOXACILLIN
e22..	FLUCLOXACILLIN
e23..	METHICILLIN SODIUM
e24..	TEMOCILLIN
e3...	BROAD SPECTRUM PENICILLINS
e31..	AMOXICILLIN
e32..	AMPICILLIN
e33..	AMPICILLIN + PENICILLINASE-RESISTANT PENICILLIN
e34..	BACAMPICILLIN HYDROCHLORIDE
e35..	*CICLACILLIN
e36..	*MEZLOCILLIN
e37..	PIPERACILLIN
e38..	PIVAMPICILLIN
e39..	TALAMPICILLIN HYDROCHLORIDE
e3A..	AMOXICILLIN [2]
e3z..	AMOXICILLIN [GENERIC ADDITIONS]
e4...	ANTIPSEUDOMONAL PENICILLINS
e41..	AZLOCILLIN
e42..	CARBENICILLIN
e43..	CARFECILLIN SODIUM
e44..	TICARCILLIN

e5...	OTHER PENICILLINS
e51..	MECILLINAM
e52..	PIVMECILLINAM HYDROCHLORIDE
e6...	CEPHALOSPORINS & CEPHAMYCINS
e61..	CEFACLOR
e62..	CEFADROXIL
e63..	CEFOTAXIME
e64..	CEFOXITIN
e65..	*CEFSULODIN SODIUM
e66..	CEFTAZIDIME
e67..	CEFTIZOXIME
e68..	CEFUROXIME
e69..	CEFALEXIN
e6a..	*CEPHALOTHIN
e6b..	CEFAMANDOLE
e6c..	CEFAZOLIN
e6d..	CEFRADINE
e6e..	*LATAMOXEF DISODIUM
e6f..	AZTREONAM
e6g..	IMIPENEM+CILASTATIN
e6h..	CEFIXIME
e6j..	CEFODIZIME
e6k..	CEFPODOXIME PROXETIL
e6m..	CEFTRIAXONE
e6n..	CEFTIBUTEN
e6o..	CEFPIROME
e6p..	CEFPROZIL
e6q..	CEFTAROLINE FOSAMIL
e6r..	CEFTOBIPROLE
e7...	TETRACYCLINES [SYSTEMIC]
e71..	TETRACYCLINE [SYSTEMIC]
e72..	CHLORTETRACYCLINE HYDROCHLORIDE [SYSTEMIC]
e73..	*CLOMOCYCLINE SODIUM
e74..	DEMECLOCYCLINE HYDROCHLORIDE
e75..	DOXYCYCLINE
e76..	LYMECYCLINE
e77..	MINOCYCLINE
e78..	OXYTETRACYCLINE
e79..	COMPOUND TETRACYCLINE PREPARATIONS
e8...	AMINOGLYCOSIDES
e81..	GENTAMICIN [INFECTIONS]
e82..	AMIKACIN
e83..	FRAMYCETIN SULFATE [SYSTEMIC]
e84..	KANAMYCIN
e85..	NEOMYCIN SULFATE [SYSTEMIC]

e86..	NETILMICIN
e87..	TOBRAMYCIN [SYSTEMIC]
e9...	MACROLIDES
e91..	ERYTHROMYCIN
e92..	CLARITHROMYCIN
e93..	AZITHROMYCIN DIHYDRATE
e94..	SPIRAMYCIN
e95..	ERYTHROMYCIN [2]
e96..	TELITHROMYCIN
ea...	CLINDAMYCIN AND LINCOMYCIN
ea1..	CLINDAMYCIN
ea2..	*LINCOMYCIN
eb...	OTHER ANTIBIOTICS
eb1..	ACROSOXACIN
eb2..	CHLORAMPHENICOL [SYSTEMIC]
eb3..	COLISTIN
eb4..	SODIUM FUSIDATE
eb5..	POLYMYXIN B SULF [SYSTEMIC]
eb6..	SPECTINOMYCIN
eb7..	VANCOMYCIN
eb8..	TEICOPLANIN
eb9..	MEROPENEM
ebA..	QUINUPRISTIN+DALFOPRISTIN
ebB..	LINEZOLID
ebC..	ERTAPENEM
ebD..	DAPTOMYCIN
ebE..	TIGECYCLINE
ebF..	DORIPENEM
ebG..	RIFAXIMIN
ebH..	FIDAXOMICIN
ebI..	FOSFOMYCIN
ebJ..	TELAVANCIN
ebK..	TEDIZOLID
ec...	SULFONAMIDES & TRIMETHOPRIM
ec1..	CO-TRIMOXAZOLE [GENERIC ADDITIONS]
ec2..	CO-TRIMOXAZOLE - (PROPRIETARY)
ec3..	*CALCIUM SULPHALOXATE
ec4..	*PHTHALYLSULPHATHIAZOLE
ec5..	SULFAMETOPYRAZINE
ec6..	SULFADIAZINE
ec7..	*SULPHADIMETHOXINE
ec8..	SULFADIMIDINE
ec9..	*SULPHAFURAZOLE
eca..	SULFAGUANIDINE
ecb..	*SULPHAUREA

ecc..	TRIMETHOPRIM
ed...	ANTITUBERCULOUS DRUGS
ed1..	CAPREOMYCIN
ed2..	CYCLOSERINE
ed3..	ETHAMBUTOL HYDROCHLORIDE
ed4..	ISONIAZID
ed5..	PYRAZINAMIDE
ed6..	RIFAMPICIN
ed7..	STREPTOMYCIN
ed8..	RIFABUTIN
ed9..	BEDAQUILINE
edA..	DELAMANID
edB..	PARA-AMINOSALICYLIC ACID
ee...	ANTILEPROTIC DRUGS
ee1..	DAPSONE
ee2..	*CLOFAZIMINE
ef...	METRONIDAZOLE & TINIDAZOLE
ef1..	METRONIDAZOLE
ef2..	TINIDAZOLE
eg...	URINARY ANTIMICROBIAL DRUGS
eg1..	NITROFURANTOIN
eg2..	CINOXACIN
eg3..	METHENAMINE
eg4..	NALIDIXIC ACID
eg5..	NOXYTIOLIN
eg6..	CIPROFLOXACIN
eg7..	ENOXACIN
eg8..	OFLOXACIN
eg9..	NORFLOXACIN
ega..	*TEMAFLOXACIN
egA..	FOSFOMYCIN TROMETAMOL
egB..	GREPAFLOXACIN
egC..	LEVOFLOXACIN
egD..	MOXIFLOXACIN
eh...	ANTIFUNGAL DRUGS
eh1..	AMPHOTERICIN [ANTI FUNGAL]
eh2..	FLUCYTOSINE
eh3..	GRISEOFULVIN
eh4..	KETOCONAZOLE [INFECTIONS]
eh5..	MICONAZOLE [SYSTEMIC]
eh6..	NATAMYCIN [SYSTEMIC]
eh7..	NYSTATIN [SYSTEMIC]
eh8..	FLUCONAZOLE
eh9..	ITRACONAZOLE
eha..	TERBINAFINE HYDROCHLORIDE [1]

ehb..	CASPOFUNGIN
ehc..	VORICONAZOLE
ehd..	POSACONAZOLE
ehe..	ANIDULAFUNGIN
ehf..	MICAFUNGIN
ei...	ANTIVIRAL DRUGS
ei1..	ACICLOVIR [SYSTEMIC]
ei2..	AMANTADINE HYDROCHLORIDE [ANTI VIRAL]
ei3..	INOSINE PRANOBEX
ei4..	*VIDARABINE [SYSTEMIC]
ei5..	ZIDOVUDINE
ei6..	RIBAVIRIN
ei7..	GANCICLOVIR
ei8..	FOSCARNET
ei9..	FAMCICLOVIR
eia..	EMTRICITABINE
eiA..	DIDANOSINE
eib..	ATAZANAVIR
eiB..	ZALCITABINE
eic..	FOSAMPRENAVIR
eiC..	VALACICLOVIR
eid..	ABACAVIR+LAMIVUDINE
eiD..	LAMIVUDINE
eie..	EMTRICITABINE+TENOFOVIR
eiE..	STAVUDINE
eif..	TIPRANAVIR
eiF..	RITONAVIR
eig..	ENTECAVIR
eiG..	SAQUINAVIR
eih..	DARUNAVIR
eiH..	INDINAVIR
eii..	TELBIVUDINE
eiI..	CIDOFOVIR
eij..	MARAVIROC
eiJ..	LAMIVUDINE+ZIDOVUDINE
eik..	EFAVIRENZ/EMTRICITABINE/TENOFOVIR
eiK..	NEVIRAPINE
eil..	RALTEGRAVIR
eiL..	NELFINAVIR
eim..	ETRAVIRINE
eiM..	IMIQUIMOD
ein..	BOCEPREVIR
eiN..	LEDIPASVIR+SOFOSBUVIR
eio..	TELAPREVIR
eiO..	EFAVIRENZ

eip..	RILPIVIRINE
eiP..	ABACAVIR
eiq..	EMTRICITABINE/RILPIVIRINE/TENOFOVIR
eiQ..	ZANAMIVIR
eir..	ELVITEGRAVIR+COBICISTAT+EMTRICITABINE+TENOFOVIR
eiR..	PALIVIZUMAB
eis..	ELVITEGRAVIR
eiS..	AMPRENAVIR
eit..	DOLUTEGRAVIR
eiT..	ABACAVIR+LAMIVUDINE+ZIDOVUDINE
eiu..	SOFOSBUVIR
eiU..	LOPINAVIR+RITONAVIR
eiv..	COBICISTAT
eiV..	TENOFOVIR
eiw..	DACLATASVIR
eiW..	VALGANCICLOVIR
eix..	SIMEPREVIR
eiX..	OSELTAMIVIR
eiy..	DOLUTEGRAVIR+ABACAVIR+LAMIVUDINE
eiY..	ADEFOVIR
eiz..	DARUNAVIR+COBICISTAT
eiZ..	ENFUVIRTIDE
ej...	ANTIMALARIALS
ej1..	*AMODIAQUINE
ej2..	CHLOROQUINE [ANTI-MALARIAL]
ej3..	HYDROXYCHLOROQUINE SULFATE [ANTI MALARIAL]
ej4..	PRIMAQUINE
ej5..	PROGUANIL HYDROCHLORIDE
ej6..	PYRIMETHAMINE
ej7..	QUININE
ej8..	MEFLOQUINE HYDROCHLORIDE
ej9..	HALOFANTRINE HYDROCHLORIDE
ejA..	MEPACRINE HYDROCHLORIDE
ejB..	ATOVAQUONE+PROGUANIL HYDROCHLORIDE
ejC..	PROGUANIL HYDROCHLORIDE+CHLOROQUINE PHOSPHATE
ejD..	ARTEMETHER+LUMEFANTRINE
ejE..	PIPERAQUINE+DIHYDROARTEMISININ
ek...	AMOEBICIDES
ek1..	DILOXANIDE FUROATE
el...	TRICHOMONACIDES
el1..	NIMORAZOLE
em...	ANTIGIARDIAL DRUGS
em1..	*ATEBRINE
en...	LEISHMANIACIDES
en1..	SODIUM STIBOGLUCONATE

Wilkinson T, *et al. J Epidemiol Community Health* 2022; 76:223–229. doi: 10.1136/jech-2021-217090

eo...	*TRYPANOCIDES[no drugs here]
ep...	DRUGS FOR TOXOPLASMOSIS [no drugs here]
eq...	DRUGS FOR THREADWORMS
eq1..	MEBENDAZOLE
eq2..	PIPERAZINE
er...	ANTHELMINTICS - ASCARICIDES
er1..	PYRANTEL
es...	ANTHELMINTICS - ANTICESTODALS
es1..	NICLOSAMIDE
es2..	ALBENDAZOLE
et...	DRUGS FOR HOOKWORMS
et1..	BEPHENIUM
eu...	SCHISTOSOMACIDES[no drugs here]
ev...	*FILARICIDES
ev1..	*DIETHYLCARBAMAZINE CITRATE
ew...	DRUGS FOR GUINEA WORMS [no drugs here]
ex...	DRUGS FOR STRONGYLOIDIASIS
ex1..	TIABENDAZOLE
ey...	PNEUMOCYSTIS PNEUMONIA DRUGS
ey1..	PENTAMIDINE ISETHIONATE
ey2..	ATOVAQUONE
ey3..	TRIMETREXATE GLUCURONATE
ez...	ANTIVIRAL DRUGS 2
ez1..	DASABUVIR
ez2..	OMBITASVIR+PARITAPREVIR+RITONAVIR
ez3..	ATAZANAVIR+COBICISTAT
f....	ENDOCRINE DRUGS
f1...	SHORT-ACTING INSULIN PREPARATIONS
f11..	*SOLUBLE INSULIN
f12..	SOLUBLE NEUTRAL INSULIN
f13..	INSULIN LISPRO
f14..	INSULIN ASPART
f15..	INSULIN GLULISINE
f2...	MEDIUM/LONG-ACTING INSULINS
f21..	BIPHASIC INSULIN
f22..	INSULIN ZINC SUSPENSION
f23..	INSULIN ZINC SUSPENSION - AMORPHOUS
f24..	INSULIN ZINC SUSPENSION - CRYSTALLINE
f25..	ISOPHANE INSULIN
f26..	PROTAMINE ZINC INSULIN
f27..	BIPHASIC ISOPHANE INSULIN
f28..	BIPHASIC ISOPHANE INSULIN 2
f29..	INSULIN GLARGINE
f2A..	INSULIN DETEMIR
f2B..	INSULIN DEGLUDEC

Wilkinson T, *et al. J Epidemiol Community Health* 2022; 76:223–229. doi: 10.1136/jech-2021-217090

f2C..	INSULIN DEGLUDEC + LIRAGLUTIDE
f3...	SULFONYLUREAS
f31..	*ACETOHEXAMIDE
f32..	CHLORPROPAMIDE
f33..	GLIBENCLAMIDE
f34..	*GLIBORNURIDE
f35..	GLICLAZIDE
f36..	GLIPIZIDE
f37..	GLIQUIDONE
f38..	*GLYMIDINE
f39..	TOLAZAMIDE
f3a..	TOLBUTAMIDE
f3A..	GLIMEPIRIDE
f4...	BIGUANIDES
f41..	METFORMIN HYDROCHLORIDE
f5...	GUAR
f51..	GUAR GUM
f6...	DIABETIC KETOACIDOSIS [no drugs here]
f7...	HYPOGLYCAEMIA TREATMENT
f71..	DIAZOXIDE [ENDOCRINE USE]
f72..	GLUCAGON
f73..	GLUCOSE [ENDOCRINE]
f8...	DIABETIC NEUROPATHY TREATMENT [no drugs here]
f9...	THYROID HORMONES
f91..	LIOTHYRONINE SODIUM
f92..	LEVOTHYROXINE SODIUM
fa...	ANTITHYROID DRUGS
fa1..	CARBIMAZOLE
fa2..	IODINE
fa3..	PROPYLTHIOURACIL
fb...	CORTICOSTEROID REPLACEMENT THERAPY
fb1..	FLUDROCORTISONE ACETATE
fb2..	DEOXYCORTONE PIVALATE [no preparations]
fc...	STEROID DISEASE SUPPRESSION [no drugs here]
fd...	CORTICOSTEROID DISADVANTAGES [no drugs here]
fe...	CORTICOSTEROID CLINICAL USE
fe1..	BETAMETHASONE
fe2..	CORTISONE ACETATE
fe3..	DEXAMETHASONE [ENDOCRINE]
fe4..	HYDROCORTISONE
fe5..	METHYLPREDNISOLONE [ENDOCRINE]
fe6..	PREDNISOLONE [ENDOCRINE]
fe7..	PREDNISONE
fe8..	TRIAMCINOLONE [ENDOCRINE]
fe9..	DEFLAZACORT

ff...	OESTROGENS
ff1..	ETHINYLESTRADIOL [ENDOCRINE]
ff2..	ESTRADIOL
ff3..	ESTRIOL [ENDOCRINE]
ff4..	CONJUGATED OESTROGENS [ENDOCRINE]
ff5..	PIPERAZINE ESTRONE SULFATE
ff6..	*QUINESTRADOL
ff7..	*QUINESTROL
ff8..	TIBOLONE
ff9..	MORE ESTRADIOL 1
fg...	PROGESTOGENS
fg1..	ALLYLOESTRENOL
fg2..	DYDROGESTERONE
fg3..	HYDROXYPROGESTERONE CAPROATE
fg4..	MEDROXYPROGEST AC [ENDOCRINE]
fg5..	NORETHISTERONE [ENDOCRINE]
fg6..	PROGESTERONE
fh...	COMBINED OESTROGEN + PROGESTOGEN PREPARATIONS
fh1..	MENSTRUAL SYMPTOMS - COMBINED PREPARATIONS
fh2..	MENSTRUAL DISORDER COMB.PREPS
fi...	MALE SEX HORMONES+ANTAGONISTS
fi1..	CYPROTERONE ACETATE [ENDOCRINE]
fi2..	MESTEROLONE
fi3..	*METHYLTESTOSTERONE
fi4..	TESTOSTERONE
fi5..	TESTOSTERONE ESTERS
fi6..	FINASTERIDE
fi7..	DUTASTERIDE
fj...	ANABOLIC STEROIDS
fj1..	NANDROLONE [ENDOCRINE]
fj2..	STANOZOLOL [ENDOC]
fk...	HYPOTHALAMIC + ANTERIOR PITUITARY HORMONES
fk1..	CHORIONIC GONADOTROPHIN
fk2..	CLOMIFENE CITRATE
fk3..	CORTICOTROPIN (ACTH)
fk4..	*CYCLOFENIL
fk5..	FOLLICLE-STIMULATING HORMONE
fk6..	*SOMATREM
fk7..	TETRACOSACTIDE
fk8..	SOMATROPIN
fk9..	SERMORELIN ACETATE
fkA..	SOMATROPIN [2]
fkB..	LUTROPIN ALFA
fkC..	FOLLITROPIN [2]
fkD..	MECASERMIN

fkE..	CORIFOLLITROPIN ALFA
fkF..	HUMAN MENOPAUSAL GONADOTROPHINS
fl...	POSTERIOR PITUITARY HORMONES
fl1..	DESMOPRESSIN
fl2..	LYPRESSIN
fl3..	*PITUITARY (POSTERIOR LOBE)
fl4..	TERLIPRESSIN
fl5..	VASOPRESSIN
fm...	ANTIDIURETIC HORMONE ANTAGON. [no drugs here]
fn...	CALCITONIN AND CALCITONIN(SALMON)
fn1..	CALCITONIN
fn2..	CALCITONIN(SALMON)
fo...	BISPHOSPHONATES
fo1..	DISODIUM ETIDRONATE
fo2..	PAMIDRONATE DISODIUM
fo3..	SODIUM CLODRONATE
fo4..	ALENDRONIC ACID
fo5..	DISODIUM TILUDRONATE
fo6..	RISEDRONATE SODIUM
fo7..	ZOLEDRONIC ACID
fo8..	IBANDRONIC ACID
fp...	BROMOCRIPTINE [ENDOCRINE]
fp1..	BROMOCRIPTINE [ENDOCRINE]
fq...	ENDOCRINE DIAGNOSTIC AGENTS
fq1..	GONADORELIN
fq2..	METYRAPONE
fq3..	PROTIRELIN
fq4..	*THYROTROPHIN
fq5..	THYROTROPIN ALFA
fr...	ENDOMETRIOSIS DRUGS
fr1..	DANAZOL
fr2..	GESTRINONE
fr3..	NAFARELIN ACETATE
fr4..	BUSERELIN ACETATE [ENDOMETRIOSIS]
fs...	TRILOSTANE
fs1..	TRILOSTANE
ft...	OTHER DRUGS USED IN DIABETES
ft1..	ACARBOSE
ft2..	TROGLITAZONE
ft3..	REPAGLINIDE
ft4..	ROSIGLITAZONE
ft5..	PIOGLITAZONE
ft6..	NATEGLINIDE
ft7..	METFORMIN + PIOGLITAZONE
ft8..	SITAGLIPTIN

ft9..	EXENATIDE
fta..	VILDAGLIPTIN
ftb..	METFORMIN + VILDAGLIPTIN
ftc..	LIRAGLUTIDE
ftd..	SAXAGLIPTIN
fte..	METFORMIN + SITAGLIPTIN
ftf..	LINAGLIPTIN
ftg..	METFORMIN + LINAGLIPTIN
fth..	DAPAGLIFLOZIN
fti..	METFORMIN + SAXAGLIPTIN
ftj..	LIXISENATIDE
ftk..	ALOGLIPTIN
ftl..	METFORMIN + ALOGLIPTIN
ftm..	METFORMIN + DAPAGLIFLOZIN
ftn..	CANAGLIFLOZIN
fto..	EMPAGLIFLOZIN
ftp..	METFORMIN + CANAGLIFLOZIN
ftq..	DULAGLUTIDE
ftr..	METFORMIN + EMPAGLIFLOZIN
fu...	OTHER ENDOCRINE DRUGS
fu1..	CABERGOLINE
fu2..	QUINAGOLIDE
fu3..	TERIPARATIDE
fu4..	PEGVISOMANT
fu5..	STRONTIUM
fu6..	CINACALCET
fu7..	PARATHYROID HORMONE
fu8..	TOLVAPTAN
fu9..	DENOSUMAB
fv...	SELECTIVE OESTROGEN RECEPTOR MODULATOR
fv1..	RALOXIFENE HYDROCHLORIDE
fw...	SHORT WITH INTERMEDIATE-ACTING INSULINS
fw1..	BIPHASIC ISOPHANE INSULIN LISPRO
fw2..	BIPHASIC INSULIN ASPART
fz...	LUTEINISING HORMONE RELEASING HORMONE ANTAGONIST PRODUCT
fz1..	CETRORELIX
fz2..	GANIRELIX
g....	OBS/GYNAE/UTI DRUGS
g1...	PROSTAGLANDINS & OXYTOCICS
g11..	DINOPROST
g12..	DINOPROSTONE
g13..	ERGOMETRINE MALEATE
g14..	GEMEPROST
g15..	OXYTOCIN
g16..	ALPROSTADIL

Wilkinson T, *et al. J Epidemiol Community Health* 2022; 76:223–229. doi: 10.1136/jech-2021-217090

g17..	CARBOPROST
g18..	MIFEPRISTONE
g19..	CARBETOCIN
g2...	MYOMETRIAL RELAXANTS
g21..	ISOXSUPRINE HYDROCHLORIDE [GYNAECOLOGICAL USE]
g22..	ORCIPRENALINE SULPHATE [OBSTETRIC USE]
g23..	RITODRINE HYDROCHLORIDE
g24..	SALBUTAMOL [OBSTETRIC]
g25..	TERBUTALINE SULFATE [MYOMETRIAL RELAXANT]
g26..	ATOSIBAN
g3...	VAGINAL TOPICAL HORMONES
g31..	CONJUG OESTROGEN [TOPICAL VAGINAL]
g32..	DIENESTROL
g33..	ESTRIOL [GYNAECOLOGICAL USE]
g34..	DIETHYLSTILBESTROL [GYNAECOLOGICAL USE]
g35..	ESTRADIOL [GYNAE]
g4...	GENITAL ANTIFUNGAL PREPARATIONS
g41..	AMPHOTERICIN [GYNAECOLOGICAL USE]
g42..	CLOTRIMAZOLE
g43..	ECONAZOLE NITRATE [GYNAECOLOGICAL USE]
g44..	ISOCONAZOLE NITRATE
g45..	KETOCONAZOLE [GYNAECOLOGICAL USE]
g46..	MICONAZOLE [GENITAL]
g47..	NATAMYCIN [GYNAECOLOGICAL USE]
g48..	NYSTATIN [GYNAECOLOGICAL USE]
g49..	FENTICONAZOLE
g5...	VAGINAL ANTIBACTERIAL PREPARATIONS
g51..	MIXED ANTIBACTERIAL VAGINAL PREPARATIONS
g52..	CLINDAMYCIN PHOSPHATE
g53..	METRONIDAZOLE [VAGINAL]
g6...	VAGINAL ANTISEPTIC PREPARATIONS
g61..	ACETIC ACID
g62..	POVIDONE-IODINE [GYNAECOLOGICAL USE]
g63..	*HYDRAGAPHEN
g7...	VAGINAL INFECTIONS OTHER PREPS
g71..	*LACTOBACILL ACIDOPHIL VACCINE
g8...	COMBINED ORAL CONTRACEPTIVE - ETHINYLOESTRADIOL
g81..	DESOGESTREL
g82..	ETYNODIOL DIACETATE [COMBINED ORAL CONTRACEPTIVE]
g83..	LEVONORGESTREL [COMBINED ORAL CONTRACEPTIVE]
g84..	*LYNOESTRENOL
g85..	NORGESTREL [COMBINED ORAL CONTRACEPTIVE]
g86..	NORETHISTERONE [COMBINED ORAL CONTRACEPTIVE]
g87..	NORETHISTERONE ACETATE [COMBINED ORAL CONTRACEPTIVE]
g88..	GESTODENE [COMBINED ORAL CONTRACEPTIVE]

g89..	NORGESTIMATE [COMBINED OC]
g8A..	DROSPIRENONE
g9...	COMBINED ORAL CONTRACEPTIVE - MESTRANOL
g91..	NORETHISTERONE [COMBINED ORAL CONTRACEPTIVE]
ga...	PROGESTOGEN ONLY CONTRACEPTIVE
gA...	PERITONEAL DIALYSIS SOLUTIONS
ga1..	ETYNODIOL DIACETATE [PROGESTOGEN ORAL CONTRACEPTIVE]
gA1..	AMINO ACIDS PERITONEAL DIALYSIS SOLUTION
ga2..	LEVONORGESTREL [PROGESTOGEN CONTRACEPTIVE]
gA2..	LOW GLUCOSE CONCENTRATION PERITONEAL DIALYSIS SOLUTION
ga3..	MEDROXYPROGESTERONE ACETATE [PROGESTOGEN ORAL CONTRACEPTIVE]
gA3..	MEDIUM GLUCOSE CONCENTRATION PERITONEAL DIALYSIS SOLUTION
ga4..	NORGESTREL [PROGESTOGEN ORAL CONTRACEPTIVE]
gA4..	HIGH GLUCOSE CONCENTRATION PERITONEAL DIALYSIS SOLUTION
ga5..	NORETHISTERONE [PROGESTOGEN ORAL CONTRACEPTIVE]
gA5..	ICODEXTRIN PERITONEAL DIALYSIS SOLUTION
ga6..	NORETHISTERONE OENANTHATE
ga7..	ETONOGESTREL
ga8..	DESOGESTREL [PROGESTOGEN ONLY CONTRACEPTIVE]
ga9..	ULIPRISTAL ACETATE
gb...	SPERMICIDAL CONTRACEPTIVES
gb1..	NONOXINOL
gb2..	OCTOXINOL
gb3..	P-DI-ISOBUTYLPHENOXYPOLYETHOXYETHANOL
gc...	URINARY RETENTION DRUGS
gc1..	BETHANECHOL CHLORIDE
gc2..	CARBACHOL [URINE RETENTION]
gc3..	*DISTIGMINE BROMIDE [URINARY]
gc4..	PHENOXYBENZAMINE HYDROCHLORIDE [URINARY RETENTION USE]
gc5..	TERAZOSIN HYDROCHLORIDE [see chap b for generic preps]
gc6..	ALFUZOSIN HYDROCHLORIDE
gc7..	TAMSULOSIN HYDROCHLORIDE
gc8..	DUTASTERIDE+TAMSULOSIN HYDROCHLORIDE
gc9..	SOLIFENACIN+TAMSULOSIN
gd...	ENURESIS FREQUENCY INCONTINENCE
gd1..	AMITRIPTYLINE HCL [ENURESIS - no drugs here]
gd2..	*EMEPRONIUM BROMIDE
gd3..	EPHEDRINE HCL [ENURESIS]
gd4..	FLAVOXATE HYDROCHLORIDE
gd5..	IMIPRAMINE HCL [ENURESIS - no drugs here]
gd6..	NORTRIPTYLINE HCL [ENURESIS - no drugs here]
gd7..	PHENYLPROPANOLAMINE HYDROCHLOR
gd8..	PROPANTHELINE BR [ENURESIS - no drugs here]
gd9..	*TERODILINE HYDROCHLORIDE
gda..	OXYBUTYNIN

Wilkinson T, *et al. J Epidemiol Community Health* 2022; 76:223–229. doi: 10.1136/jech-2021-217090

gdA..	TOLTERODINE L-TARTRATE
gdb..	COLLAGEN IMPLANTS
gdB..	TROSPIUM CHLORIDE [ENURESIS]
gdc..	PROPIVERINE HYDROCHLORIDE
gdd..	SOLIFENACIN
gde..	DULOXETINE
gdf..	DARIFENACIN
gdg..	FESOTERODINE FUMARATE
gdh..	MIRABEGRON
ge...	URINARY ANALGESIA & PH CHANGE
ge1..	ASCORBIC ACID [URINE PH - no drugs here]
ge2..	*PHENAZOPYRIDINE HYDROCHLORIDE
ge3..	POTASSIUM CITRATE
ge4..	SODIUM BICARBONATE [URINE PH]
ge5..	SODIUM CITRATE
ge6..	OTHER URINARY DISORDER PREPARATIONS
gf...	BLADDER INSTILLATIONS
gf1..	SIMPLE BLADDER INSTILLATIONS
gf2..	CATHETER PATENCY SOLUTIONS
gf3..	DIAGNOSTIC BLADDER AGENTS
gg...	VAGINAL LUBRICANTS
gg1..	PETROLEUM JELLY
gg2..	POLYCARBOPHIL
gg3..	AQUEOUS LUBRICANTS
gh...	TREATMENT OF SEXUAL DYSFUNCTION AND IMPOTENCE
gh1..	YOHIMBINE/PEMOLINE/METHYLTESTOSTERONE
gh2..	ALPROSTADIL [ERECTILE DYSFUNCTION]
gh3..	PAPAVERINE
gh4..	MOXISYLYTE HCL [ERECTILE DYSFUNCT]
gh5..	SILDENAFIL
gh6..	APOMORPHINE HYDROCHLORIDE [ERECTILE DYSFUNCTION]
gh7..	TADALAFIL
gh8..	VARDENAFIL
gh9..	YOHIMBINE
ghA..	AVANAFIL
gi...	VAGINAL PH IMBALANCE
gi1..	LACTIC ACID
gi2..	POLYSACCHARIDE COMPLEX
gj...	CONTRACEPTIVE IMPLANT
gj1..	COMBINED CONTRACEPTIVE IMPLANT
gk...	COMBINED ORAL CONTRACEPTIVE - ESTRADIOL VALERATE
gk1..	DIENOGEST (COMBINED ORAL CONTRACEPTIVE)
gl...	COMBINED ORAL CONTRACEPTIVE - ESTRADIOL
gl1..	NOMEGESTROL ACETATE (COMBINED ORAL CONTRACEPTIVE)
gm...	PREMATURE EJACULATION

gm1..	DAPOXETINE
h....	CHEMOTHERAPY/IMMUNOSUPPRESSANT DRUGS
h1...	ALKYLATING DRUGS
h11..	BUSULFAN
h12..	CARMUSTINE
h13..	CHLORAMBUCIL
h14..	CYCLOPHOSPHAMIDE
h15..	ESTRAMUSTINE PHOSPHATE
h16..	*ETHOGLUCID
h17..	IFOSFAMIDE
h18..	LOMUSTINE
h19..	MELPHALAN
h1a..	MITOBRONITOL
h1b..	*MUSTINE HYDROCHLORIDE
h1c..	THIOTEPA
h1d..	TREOSULFAN
h1e..	BENDAMUSTINE HYDROCHLORIDE
h2...	CYTOTOXIC ANTIBIOTICS
h21..	ACTINOMYCIN D
h22..	BLEOMYCIN
h23..	DOXORUBICIN HYDROCHLORIDE
h24..	EPIRUBICIN HYDROCHLORIDE
h25..	MITOMYCIN
h26..	PLICAMYCIN
h27..	IDARUBICIN HYDROCHLORIDE
h28..	*ACLARUBICIN
h29..	DAUNORUBICIN
h3...	ANTIMETABOLITES
h31..	CYTARABINE
h32..	FLUOROURACIL
h33..	MERCAPTOPURINE
h34..	METHOTREXATE
h35..	TIOGUANINE
h36..	GEMCITABINE
h38..	MERCURIC OXIDE [ANTIMETABOLITE]no drugs here
h39..	RALTITREXED
h3A..	CAPECITABINE
h3B..	TEGAFUR+URACIL
h3C..	PEMETREXED
h3D..	CLOFARABINE
h3E..	NELARABINE
h3F..	AZACITIDINE
h3G..	METHOTREXATE(2)
h3H..	TEGAFUR
h3I..	DECITABINE

h4...	VINCA ALKALOIDS + ETOPOSIDE
h41..	ETOPOSIDE
h42..	VINBLASTINE SULFATE
h43..	VINCRISTINE SULFATE
h44..	VINDESINE SULFATE
h45..	VINORELBINE
h46..	VINFLUNINE DITARTRATE
h5...	OTHER CYTOXIC DRUGS
h51..	AMSACRINE
h52..	CARBOPLATIN
h53..	CISPLATIN
h54..	DACARBAZINE
h55..	HYDROXYCARBAMIDE
h56..	MITOXANTRONE
h57..	PROCARBAZINE
h58..	RAZOXANE
h59..	CRISANTASPASE
h5A..	PENTOSTATIN
h5B..	PACLITAXEL
h5C..	FLUDARABINE
h5D..	CLADRIBINE
h5E..	DOCETAXEL
h5F..	TOPOTECAN
h5G..	IRINOTECAN HYDROCHLORIDE TRIHYDRATE
h5H..	ALTRETAMINE
h5I..	OXALIPLATIN
h5J..	TEMOZOLOMIDE
h5K..	VERTEPORFIN
h5L..	METHYL AMINOLEVULINATE
h5M..	PIXANTRONE
h6...	ANTAGONISTS + CYTOTOXICS
h61..	MESNA
h62..	DEXRAZOXANE
h7...	CYTOTOXIC IMMUNOSUPPRESSANTS
h71..	AZATHIOPRINE [IMMUNOSUPPRESSANT]
h8...	OTHER IMMUNOSUPPRESSANTS
h81..	ANTILYMPHOCYTE IMMUNOGLOBULIN
h82..	CICLOSPORIN
h83..	TACROLIMUS
h84..	MYCOPHENOLATE
h85..	SIROLIMUS
h86..	ANAKINRA
h87..	ADALIMUMAB
h88..	EFALIZUMAB
h89..	ETANERCEPT

h8A..	NATALIZUMAB
h8B..	INFLIXIMAB
h8C..	ABATACEPT
h8D..	ANTITHYMOCYTE IMMUNOGLOBULIN
h8E..	USTEKINUMAB
h8F..	TOCILIZUMAB
h8G..	CERTOLIZUMAB PEGOL
h8H..	CANAKINUMAB
h8I..	GOLIMUMAB
h8J..	FINGOLIMOD
h8K..	BELIMUMAB
h8L..	BELATACEPT
h8M..	PIRFENIDONE
h8N..	POMALIDOMIDE
h8O..	SILTUXIMAB
h8P..	VEDOLIZUMAB
h8Q..	APREMILAST
h8R..	SECUKINUMAB
h9...	IMMUNOSTIMULANTS
h91..	CORYNEBACTERIUM PARVUM VACCINE
h92..	MOLGRAMOSTIM
h93..	LEVAMISOLE HCL [IMMUNOSTIMULANT]
h94..	LENOGRASTIM
h95..	AMIFOSTINE
h96..	PLERIXAFOR
h97..	MIFAMURTIDE
h98..	HISTAMINE
ha...	INTERFERONS
ha1..	INTERFERONS
ha2..	INTERFERONS (2)
ha3..	INTERFERONS (3)
ha5..	DACLIZUMAB
hb...	OESTROGENS IN MALIGNANCY
hb1..	ETHINYLOESTRADIOL [MALIGNANCY]
hb2..	FOSFESTROL TETRASODIUM
hb3..	POLYESTRADIOL PHOSPHATE
hb4..	DIETHYLSTILBESTROL [MALIGNANCY]
hc...	PROGESTOGENS IN MALIGNANCY
hc1..	GESTONORONE HEXANOATE
hc2..	MEDROXYPROGEST AC [MALIGNANCY]
hc3..	MEGESTROL ACETATE
hc4..	NORETHISTERONE [MALIGNANCY]
hc5..	NORETHISTERONE AC [MALIGNANCY]
hd...	ANDROGENS + ANABOLIC STEROIDS
hd1..	*DROSTANOLONE PROPIONATE

hd2..	NANDROLONE [malignancy] [no preps here]
he...	HORMONE ANTAGONISTS
he1..	AMINOGLUTETHIMIDE
he2..	BUSERELIN
he3..	CYPROTERONE ACET [MALIGNANCY]
he4..	TAMOXIFEN
he5..	GOSERELIN
he6..	OCTREOTIDE ACETATE
he7..	FLUTAMIDE
he8..	LEUPRORELIN ACETATE
he9..	FORMESTANE
heA..	BICALUTUMIDE
heB..	TRIPTORELIN
heC..	ANASTROZOLE
heD..	TOREMIFENE
heE..	LETROZOLE
heF..	LANREOTIDE
heG..	EXEMESTANE
heH..	FULVESTRANT
heI..	DEGARELIX
heJ..	HISTRELIN
heK..	ABIRATERONE ACETATE
heL..	PASIREOTIDE
heM..	ENZALUTAMIDE
hf...	INTERLEUKINS
hf1..	ALDESLEUKIN
hg...	CYTOSTATIC DRUGS
hg1..	TRETINOIN [CYTOSTATIC]
hh...	OTHER ANTINEOPLASTIC AGENTS
hh1..	RITUXIMAB
hh2..	TRASTUZUMAB
hh3..	ALEMTUZUMAB
hh4..	IMATINIB
hh5..	BEXAROTENE
hh6..	TEMOPORFIN
hh7..	ARSENIC
hh8..	BORTEZOMIB
hh9..	CETUXIMAB
hha..	RUXOLITINIB
hhA..	ANAGRELIDE
hhb..	AXITINIB
hhB..	BEVACIZUMAB
hhc..	CRIZOTINIB
hhC..	ERLOTINIB
hhd..	BRENTUXIMAB VEDOTIN

hhD..	MITOTANE PRODUCT
hhe..	PERTUZUMAB
hhE..	SORAFENIB
hhf..	BOSUTINIB
hhF..	SUNITINIB
hhg..	PONATINIB
hhG..	DASATINIB
hhh..	REGORAFENIB
hhH..	LENALIDOMIDE
hhi..	VISMODEGIB
hhI..	ECULIZUMAB
hhj..	AFATINIB
hhJ..	PANITUMUMAB
hhk..	DABRAFENIB
hhK..	TRABECTEDIN
hhl..	TRASTUZUMAB EMTANSINE
hhL..	NILOTINIB
hhm..	OBINUTUZUMAB
hhM..	LAPATINIB
hhn..	IDELALISIB
hhN..	THALIDOMIDE
hho..	RAMUCIRUMAB
hhO..	TEMSIROLIMUS
hhp..	IBRUTINIB
hhP..	GEFITINIB
hhq..	NINTEDANIB
hhQ..	EVEROLIMUS
hhr..	CABOZANTINIB
hhR..	OFATUMUMAB
hhs..	OLAPARIB
hhS..	PAZOPANIB
hht..	NIVOLUMAB
hhT..	ERIBULIN MESYLATE
hhu..	LENVATINIB
hhU..	CABAZITAXEL
hhv..	PEMBROLIZUMAB
hhV..	AMINOLEVULINIC ACID
hhW..	IPILIMUMAB
hhX..	CATUMAXOMAB
hhY..	VEMURAFENIB
hhZ..	VANDETANIB
i....	HAEMATOLOGY/DIETETIC DRUGS
i1...	IRON DEFICIENCY - ORAL IRON
i11..	FERROUS FUMARATE
i12..	FERROUS GLUCONATE

i13..	FERROUS GLYCINE SULFATE
i14..	FERROUS SUCCINATE
i15..	FERROUS SULFATE
i16..	IRON & FOLIC ACID
i17..	POLYSACCHARIDE-IRON COMPLEX
i18..	SODIUM FEREDETATE
i19..	COMPOUND IRON PREPARATIONS
i1z..	IRON PRODUCT
i2...	PARENTERAL IRON THERAPY
i21..	IRON DEXTRAN COMPLEX
i22..	IRON SORBITOL COMPLEX
i23..	IRON HYDROXIDE SUCROSE COMPLEX
i24..	IRON CARBOXYMALTOSE COMPLEX
i25..	IRON ISOMALTOSIDE COMPLEX
i3...	MEGALOBLASTIC ANAEMIAS
i31..	HYDROXOCOBALAMIN
i32..	CYANOCOBALAMIN
i33..	FOLIC ACID
i34..	FOLINIC ACID
i4...	HYPOPLASTIC & HAEMOLYTIC ANAEMIA DRUGS
i41..	NANDROLONE [BLOOD]
i42..	OXYMETHOLONE
i43..	*STANOZOLOL [ANAEMIA]
i44..	EPOETIN ALFA
i45..	EPOETIN BETA
i46..	FILGRASTIM
i47..	DARBEPOETIN ALFA
i48..	PEGFILGRASTIM
i49..	EPOETIN DELTA
i4A..	METHOXY PEG-EPOETIN BETA
i4B..	EPOETIN ZETA
i4C..	EPOETIN THETA
i4D..	LIPEGFILGRASTIM
i5...	THROMBOCYTOPENIC PURPURA [no drugs here]
i6...	ELECTROLYTES-ORAL-POTASSIUM
i61..	POTASSIUM SALTS
i7...	DRUGS FOR HYPERKALAEMIA
i71..	POLYSTYRENE SULFONATE RESINS
i8...	ELECTROLYTES-ORAL-SODIUM
i81..	SODIUM SALTS
i9...	ELECTROLYTES INTRAVENOUS
i91..	GLUCOSE - INTRAVENOUS
i92..	POTASSIUM CHLORIDE I.V.
i93..	SODIUM BICARBONATE I.V.
i94..	SODIUM CHLORIDE I.V.

i95..	SODIUM LACTATE I.V.
i96..	WATER FOR INJECTION
i97..	POTASSIUM PHOSPHATE I.V
i98..	POTASSIUM ACID PHOSPHATE I.V
i99..	MAGNESIUM SULFATE I.V
ia...	PLASMA SUBSTITUTES
iA...	MINERALS - MAGNESIUM
ia1..	DEXTRAN 40 I.V.INFUSION
iA1..	MAGNESIUM ASPARTATE HYDROCHLORIDE
ia2..	DEXTRAN 70 I.V.INFUSION
ia3..	DEXTRAN 110 intravenous infusion
ia4..	GELATIN
ia5..	HETASTARCH
ia6..	PENTASTARCH
ia7..	ALBUMIN SOLUTION
ia8..	HEXASTARCH
ia9..	OTHER HYDROXYETHYL ETHER STARCH
ib...	NUTRITION - INTRAVENOUS
iB...	HAEM PRODUCTS
ib1..	PARENTERAL NUTRITION A - F
iB1..	HAEM ARGINATE
ib2..	PARENTERAL NUTRITION G - N
ib3..	PARENTERAL NUTRITION O - Z
ib4..	PARENTERAL SUPPLEMENTARY PREPARATIONS
ib5..	PARENTERAL NUTRITION G - N (2)
ic...	ORAL SUPPLEMENT
iC...	DRUGS USED IN HYPERURICAEMIA
ic1..	ORAL SUPPLEMENT
iC1..	RASBURICASE
iC2..	FEBUXOSTAT
id...	FOODS FOR SPECIAL DIETS
iD...	MINERALS - SELENIUM
id1..	GLUTEN FREE FOOD
iD1..	SELENIUM PRODUCT
id2..	GLUTEN FREE & PKU FOOD
id3..	PHENYLKETONURIA FOOD
id4..	RENAL FAILURE/KETOGEN FOOD
id5..	MILK INTOLERANCE FOOD
id6..	HYPOPROTEINAEMIA FOOD
id7..	CYSTIC FIBROSIS FOOD
id8..	*HYPERCHOLESTEROLAEMIA
id9..	GLUCOSE/GALACTOSE INTOLERANCE
ida..	GLYCOGEN STORAGE DISEASE
idA..	RENAL FAILURE/KETOGEN FOOD (2)
idb..	HOMOCYSTINURIA

idB..	PHENYLKETONURIA FOOD [3]
idc..	PROTEIN MALABSORPTION
idC..	MORE GLUTEN FREE FOODS[7]
idd..	FEED THICKENING PREPS
idD..	CROHNS DISEASE
ide..	CALCIUM INTOLERANCE FOOD
idE..	INHERITED METABOLIC DISORDERS
idf..	MAPLE SYRUP URINE DISEASE
idF..	PHENYLKETONURIA FOOD (4)
idg..	LOW SODIUM FOOD
idG..	MORE GLUTEN FREE FOODS[8]
idh..	MINERAL SUPPLEMENTS
idH..	AMINOACIDOPATHY [OTHER] FOODS (2)
idi..	CHRONIC LIVER DISEASE
idI..	PHENYLKETONURIA FOOD (5)
idj..	AMINOACIDOPATHY [OTHER] FOODS
idk..	PROTEIN INTOLERANCE FOOD
idl..	ADRENOLEUKODYSTROPHY FOODS
idm..	MORE GLUTEN FREE FOODS
idn..	ISOVALERIC ACIDAEMIA FOODS
ido..	MORE GLUTEN FREE FOODS[2]
idp..	LONG CHAIN ACYL-COA DEHYDROGENASE DEFICIENCY FOOD
idq..	HYPERCHOLESTEROLAEMIA FOOD
idr..	UREA CYCLE DISORDERS FOOD
ids..	MORE GLUTEN FREE FOODS[3]
idt..	PHENYLKETONURIA FOOD[2]
idu..	MORE GLUTEN FREE FOODS[4]
idv..	MORE GLUTEN FREE FOODS[5]
idw..	MORE GLUTEN FREE FOODS[6]
idy..	GLUTEN-FREE FOODS [GENERIC 1]
idz..	GLUTEN-FREE FOODS [GENERIC]
ie...	SUPPLEMENT & COMPLETE FEEDS
iE...	PLASMA PRODUCT
ie1..	SUPP & COMP FEEDS A - F
iE1..	C1-ESTERASE INHIBITOR
ie2..	SUPPL. & COMPLETE FEEDS F - P
iE2..	CONESTAT ALFA
ie3..	SUPPL. & COMPLETE FEEDS N - Z
ie4..	DIALYSIS FOOD SUPPLEMENT
ie5..	MORE SUPPLEMENT & COMPLETE FOODS A-Z
ie6..	MORE SUPPLEMENT & COMPLETE FOODS [1]
ie7..	MORE SUPPLEMENT & COMPLETE FOODS [2]
ie8..	MORE SUPPLEMENT & COMPLETE FOODS [3]
ie9..	MORE SUPPLEMENT & COMPLETE FOODS [4]
ieA..	MORE SUPPLEMENT & COMPLETE FOODS [5]

ieB..	MORE SUPPLEMENT & COMPLETE FOODS [6]
ieC..	MORE SUPPLEMENT & COMPLETE FOODS [7]
ieD..	MORE SUPPLEMENT & COMPLETE FOODS [8]
ieE..	MORE SUPPLEMENT & COMPLETE FOODS [9]
ieF..	MORE SUPPLEMENT & COMPLETE FOODS [10]
ieG..	MORE SUPPLEMENT & COMPLETE FOODS [11]
if...	DIETARY SUPPLEMENTS - OTHER
iF...	METHAEMOGLOBINAEMIA
if1..	ORAL SUPPLEMENT - OTHER
iF1..	METHYLTHIONINIUM CHLORIDE
ig...	MINERALS - CALCIUM SUPPLEMENTS
ig1..	CALCIUM SALTS
ih...	HYPERCALCAEMIA
ih1..	SODIUM CELLULOSE PHOSPHATE
ih2..	TRISODIUM EDETATE
ii...	MINERALS-PHOSPHATE SUPPLEMENTS
ii1..	PHOSPHATE SALTS
ij...	PHOSPHATE BINDING AGENTS
ij1..	ALUMINIUM HYDROXIDE [NUTRITIONAL USE]
ij2..	CALCIUM CARBONATE
ij3..	CALCIUM ACETATE
ij4..	SEVELAMER
ij5..	LANTHANUM
ij6..	CALCIUM ACETATE+MAGNESIUM CARBONATE
ij7..	COLESTILAN
ij8..	SUCROFERRIC OXYHYDROXIDE
ik...	MINERALS-FLUORIDE
ik1..	SODIUM FLUORIDE
ik2..	STANNOUS FLUORIDE
il...	MINERALS - ZINC
il1..	ZINC SALTS
im...	VITAMIN A
im1..	VITAMIN A
im2..	COMPOUND A & D VITAMINS
in...	VITAMIN B GROUP
in1..	NICOTINAMIDE
in2..	PYRIDOXINE HYDROCHLORIDE
in3..	RIBOFLAVIN
in4..	THIAMINE HYDROCHLORIDE
in5..	VITAMINS B & C
in6..	POTASSIUM AMINOBENZOATE
in7..	VITAMIN B COMPLEX
io...	VITAMIN C
io1..	ASCORBIC ACID [NUTRITION]
ip...	VITAMIN D

Wilkinson T, *et al. J Epidemiol Community Health* 2022; 76:223–229. doi: 10.1136/jech-2021-217090

ip1..	ALFACALCIDOL
ip2..	CALCITRIOL
ip3..	CHOLECALCIFEROL OR ERGOCALCIFEROL
ip4..	DIHYDROTACHYSTEROL
ip5..	PARICALCITOL
ip6..	COLECALCIFEROL (2)
iq...	VITAMIN E
iq1..	ALPHA TOCOPHERYL ACETATE
iq2..	D-ALPHA-TOCOPHEROL
ir...	VITAMIN K
ir1..	MENADIOL SODIUM PHOSPHATE
ir2..	PHYTOMENADIONE
is...	MULTIVITAMIN PREPARATIONS
is1..	MULTIVITAMINS
it...	BITTERS & TONICS
it1..	TONICS
it2..	*STRYCHNINE [no preparations]
iu...	DRUGS USED IN METABOLIC DISORDERS
iu1..	*PENICILLAMINE [METABOLIC]
iu2..	TRIENTINE DIHYDROCHLORIDE
iu3..	MERCAPTAMINE
iu4..	SODIUM PHENYLBUTYRATE
iu5..	MIGLUSTAT
iu6..	NITISINONE
iu7..	BETAINE
iu8..	SAPROPTERIN DIHYDROCHLORIDE
iv...	IRON POISONING
iv1..	DESFERRIOXAMINE MESYLATE
iv2..	DEFERIPRONE
iv3..	DEFERASIROX
iw...	CARNITINE DEFICIENCY PRODUCTS
iw1..	CARNITINE PRODUCTS
ix...	ENZYME DEFICIENCY PRODUCTS
ix1..	ALGLUCERASE
ix2..	IMIGLUCERASE
ix3..	AGALSIDASE BETA
ix4..	CARGLUMIC ACID
ix5..	LARONIDASE
ix6..	ALGLUCOSIDASE ALFA
ix7..	AGALSIDASE ALFA
ix8..	IDURSULFASE
ix9..	GALSULFASE
ixA..	VELAGLUCERASE ALFA
iz...	OVER THE COUNTER SUPPLEMENTARY PRODUCTS
iz1..	OVER THE COUNTER HEALTHFOOD/HERBAL PRODUCTS

Wilkinson T, *et al. J Epidemiol Community Health* 2022; 76:223–229. doi: 10.1136/jech-2021-217090

j....	MUSCULOSKELETAL DRUGS
j1...	ASPIRIN AND THE SALICYLATES
j11..	ASPIRIN [MUSCULOSKELETAL USE]
j12..	BENORILATE [MUSCULOSKELETAL USE]
j13..	CHOLINE MAGNES. TRISALICYLATE
j14..	SALSALATE
j15..	SODIUM SALICYLATE
j2...	NON-STEROIDAL ANTI-INFLAMMATORY DRUGS
j21..	AZAPROPAZONE
j22..	DICLOFENAC SODIUM
j23..	DIFLUNISAL [MUSCULOSKELETAL USE]
j24..	ETODOLAC
j25..	FENBUFEN
j26..	FENOPROFEN [MUSCULOSKELETAL USE]
j27..	FLURBIPROFEN
j28..	IBUPROFEN [MUSCULOSKELETAL USE]
j29..	INDOMETACIN
j2a..	KETOPROFEN
j2b..	MEFENAMIC ACID [MUSCULOSKELETAL USE]
j2c..	NAPROXEN
j2d..	PHENYLBUTAZONE
j2e..	PIROXICAM
j2f..	SULINDAC
j2g..	TIAPROFENIC ACID
j2h..	TOLMETIN
j2i..	PIROXICAM-BETADEX
j2j..	ACEMETACIN
j2k..	NABUMETONE
j2l..	TENOXICAM
j2m..	ACECLOFENAC
j2n..	MELOXICAM
j2o..	DICLOFENAC SODIUM 2
j2p..	IBUPROFEN [MUSCULOSKELETAL USE 2]
j2q..	DEXKETOPROFEN
j2r..	DICLOFENAC POTASSIUM
j2s..	LORNOXICAM
j2t..	DEXIBUPROFEN
j3...	SYSTEMIC STEROIDS + ACTH
j4...	LOCAL CORTICOSTEROID INJECTIONS
j41..	DEXAMETHASONE SODIUM PHOSPHATE [MUSCULOSKELETAL USE]
j42..	HYDROCORTISONE ACETATE [MUSCULOSKELETAL USE]
j43..	METHYLPREDNIS. ACETATE [MUSCULOSKELETAL USE]
j44..	PREDNISOLONE ACETATE [MUSCULOSKELETAL USE]
j45..	PREDNISOLONE SOD PHOS [MUSC-SK]
j46..	TRIAMCINOLONE ACETONIDE [MUSCULOSKELETAL USE]

j47..	TRIAMCINOLONE HEXACETONIDE
j5...	RHEUMATIC DIS. PROCESS DRUGS
j51..	SODIUM AUROTHIOMALATE
j52..	PENICILLAMINE [MUSCULOSKELETAL USE]
j53..	CHLOROQUINE [MUSCULO-SKELETAL]
j54..	HYDROXYCHLOROQUINE SULFATE [ANTI-RHEUMATIC]
j55..	SULFASALAZINE [MUSCULOSKELETAL USE]
j56..	AURANOFIN
j57..	HYLAN
j58..	SODIUM HYALURONATE [ANTI-RHEUMATIC]
j59..	LEFLUNOMIDE
j6...	GOUT DRUGS
j61..	ALLOPURINOL
j62..	COLCHICINE
j63..	PROBENECID
j64..	SULFINPYRAZONE
j7...	NEUROMUSCULAR TRANSMISSION DRUG
j71..	DISTIGMINE BROMIDE [MUSCULOSKELETAL USE]
j72..	EDROPHONIUM CHLOR [NEUROMUSCULAR TRANSMISSION]
j73..	NEOSTIGMINE [NEUROMUSCULAR TRANSMISSION]
j74..	PYRIDOSTIGMINE BROMIDE
j75..	AMIFAMPRIDINE
j8...	SKELETAL MUSCLE RELAXANTS
j81..	DIAZEPAM [SKELETAL MUSCLE RELAXANT]
j82..	BACLOFEN
j83..	*CARISOPRODOL
j84..	DANTROLENE SOD [MUSCULOSKELETAL USE]
j85..	METHOCARBAMOL
j86..	ORPHENADRINE CITRATE
j87..	TIZANIDINE
j9...	ENZYMES - SOFT TISSUE INFLAMMATION RELIEF
j91..	*BROMELAINS
j92..	CHYMOTRYPSIN [MUSCULOSKELETAL USE]
j93..	*DEOXYRIBONUCLEASE
j94..	HYALURONIDASE
ja...	RUBEFACIENTS + TOPICAL ANTIRHEUMATICS
jA...	CYCLO-OXYGENASE-2 INHIBITOR DRUG
ja1..	TOPICAL ANTI-INFLAMMATORY PREP[1]
jA1..	*ROFECOXIB
ja2..	TOPICAL ANTI-INFLAMMATORY PREP[2]
jA2..	CELECOXIB
ja3..	TOPICAL ANTI-INFLAMMATORY PREP[3]
jA3..	PARECOXIB
jA5..	ETORICOXIB
jA6..	*VALDECOXIB

Wilkinson T, *et al. J Epidemiol Community Health* 2022; 76:223–229. doi: 10.1136/jech-2021-217090

jA7..	*LUMIRACOXIB
jb...	OTHER PREPS FOR INFLAMED JOINTS
jB...	BONE CEMENTS
jb1..	PARAFFIN WAX - LOW M.P.
jB1..	GENTAMICIN BONE CEMENTS
jB2..	PLAIN BONE CEMENTS
jB3..	DIBOTERMIN ALFA
jc...	SELECTIVE RELAXANT BINDING AGENT
jc1..	SUGAMMADEX
jd...	ENZYMES - DISORDER OF THE MUSCULO-SKELETAL SYSTEM
jd1..	COLLAGENASE
k....	EYE DRUGS
k1...	ADMINISTRATION OF EYE DRUGS -[no drugs here]
k2...	CONTROL OF MICROBIAL CONTAMIN.-[no drugs here]
k3...	TOPICAL PREPARATIONS [EYE]
k31..	ACICLOVIR [EYES]
k32..	CHLORAMPHENICOL [EYE]
k33..	CHLORTETRACYCLINE HCL [EYE]
k34..	FRAMYCETIN SULFATE [EYE]
k35..	GENTAMICIN [EYE]
k36..	IDOXURIDINE [EYE]
k37..	*MAFENIDE PROPIONATE
k38..	*MERCURIC OXIDE [EYE]
k39..	NEOMYCIN SULFATE [EYE]
k3a..	POLYMYXIN B SULF [EYE]
k3b..	PROPAMIDINE ISETHIONATE
k3c..	SULFACETAMIDE SODIUM
k3d..	TETRACYCLINE HCL [EYE]
k3e..	TOBRAMYCIN [EYE]
k3f..	*VIDARABINE [EYE]
k3g..	FUSIDIC ACID [EYE]
k3h..	NORFLOXACIN [EYE]
k3i..	*FOMIVIRSEN [EYE]
k3j..	OFLOXACIN [EYE]
k3k..	CIPROFLOXACIN [EYE]
k3l..	LOMEFLOXACIN
k3m..	GANCICLOVIR [EYE]
k3n..	POVIDONE IODINE
k4...	FUNGAL EYE INFECTIONS
k5...	SYSTEMIC PREPARATIONS - [no drugs here]
k6...	CORTICOSTER + ANTI-INFL [EYE]
k61..	ANTAZOLINE SALTS
k62..	BETAMETHASONE SODIUM PHOSHATE [EYE]
k63..	CLOBETASONE BUTYRATE [EYE]
k64..	DEXAMETHASONE [EYE]

k65..	FLUOROMETHOLONE
k66..	HYDROCORTISONE ACETATE [EYE]
k67..	OXYPHENBUTAZONE
k68..	PREDNISOLONE SOD PHOS [EYE]
k69..	SODIUM CROMOGLICATE [EYE]
k6a..	PREDNISOLONE ACETATE [EYE]
k6b..	LODOXAMIDE [EYE]
k6c..	FLURBIPROFEN SODIUM [EYE]
k6d..	DICLOFENAC SODIUM [EYE]
k6e..	NEDOCROMIL SODIUM [EYE]
k6f..	LEVOCABASTINE HYDROCHLORIDE [EYE]
k6g..	KETOROLAC TROMETAMOL [EYE]
k6h..	AZELASTINE HYDROCHLORIDE [EYE]
k6i..	EMEDASTINE [EYE]
k6j..	RIMEXOLONE
k6k..	KETOTIFEN [EYE]
k6l..	OLOPATADINE [EYE]
k6m..	LOTEPREDNOL ETABONATE
k6n..	NEPAFENAC
k6o..	BROMFENAC
k7...	MYDRIATICS AND CYCLOPLEGICS
k71..	ATROPINE SULFATE [EYES]
k72..	CYCLOPENTOLATE HYDROCHLORIDE
k73..	HOMATROPINE HYDROBROMIDE
k74..	HYOSCINE HYDROBROMIDE [EYE]
k75..	LACHESINE CHLORIDE
k76..	PHENYLEPHRINE HCL [EYE]
k77..	TROPICAMIDE
k78..	TROPICAMIDE+PHENYLEPHRINE
k8...	TREATMENT OF GLAUCOMA
k81..	ACETAZOLAMIDE [GLAUCOMA]
k82..	ADRENALINE
k83..	BETAXOLOL HYDROCHLORIDE [EYE]
k84..	CARBACHOL [EYE]
k85..	CARTEOLOL HYDROCHLORIDE
k86..	DEMECARIUM BROMIDE
k87..	DICHLORPHENAMIDE
k88..	DIPIVEFRINE HYDROCHLORIDE
k89..	ECOTHIOPATE IODIDE
k8a..	GUANETHIDINE MONOSULFATE [EYE]
k8b..	METIPRANOLOL
k8c..	PHYSOSTIGMINE SULPHATE
k8d..	PILOCARPINE HYDROCHLORIDE
k8e..	PILOCARPINE NITRATE
k8f..	TIMOLOL MALEATE [EYE]

k8g..	LEVOBUNOLOL HYDROCHLORIDE
k8h..	DORZOLAMIDE
k8i..	LATANOPROST [EYE]
k8j..	BRIMONIDINE
k8k..	DORZOLAMIDE+TIMOLOL
k8l..	BRINZOLAMIDE
k8m..	LATANOPROST+TIMOLOL
k8n..	TRAVOPROST
k8o..	BIMATOPROST
k8p..	BRIMONIDINE+TIMOLOL
k8q..	TIMOLOL+TRAVOPROST
k8r..	BIMATOPROST+TIMOLOL
k8s..	BRINZOLAMIDE+TIMOLOL
k8t..	TAFLUPROST
k8u..	BRINZOLAMIDE+BRIMONIDINE
k8v..	TAFLUPROST+TIMOLOL
k9...	LOCAL ANAESTHETICS [EYE]
k91..	TETRACAINE HYDROCHLORIDE
k92..	COCAINE HYDROCHLORIDE
k93..	LIDOCAINE HYDROCHLORIDE [EYE]
k94..	OXYBUPROCAINE HYDROCHLORIDE
k95..	PROXYMETACAINE HYDROCHLORIDE
ka...	TEAR DEFICIENCY PREPARATIONS
ka1..	ACETYLCYSTEINE [EYES]
ka2..	HYPROMELLOSE
ka3..	LIQUID PARAFFIN [EYE]
ka4..	POLYVINYL ALCOHOL
ka5..	HYDROXYETHYLCELLULOSE
ka6..	CARBOMER-940
ka7..	POVIDONE
ka8..	CARBOMERS
ka9..	CARMELLOSE SODIUM [EYE]
kaA..	RETINOL PALMITATE
kaB..	SOYBEAN
kb...	OTHER EYE PREPARATIONS
kb1..	ACETYLCHOLINE CHLORIDE
kb2..	*CASTOR OIL [EYE]
kb3..	CHYMOTRYPSIN [EYE]
kb4..	PARAFFIN - SOFT YELLOW
kb5..	SODIUM CHLORIDE [EYE]
kb6..	THYMOXAMINE HYDROCHLORIDE [EYE]
kb7..	*ZINC SULPHATE [EYE]
kb8..	SODIUM HYALURONATE
kb9..	APRACLONIDINE HYDROCHLORIDE
kbA..	HYDROXYPROPYLMETHYLCELLULOSE

kbB..	PARAFFIN-SOFT WHITE [EYE]
kbC..	WITCH HAZEL PRODUCT
kbD..	PEGAPTANIB
kbE..	RANIBIZUMAB
kbF..	HYDROXYPROPYL GUAR
kbG..	AFLIBERCEPT
kc...	DIAGNOSTIC PREPARATIONS
kc1..	FLUORESCEIN SODIUM
kc2..	ROSE BENGAL
kd...	CONTACT LENSES - [no drugs here]
l....	ENT DRUGS
l1...	OTITIS EXT. - ASTRINGENT PREPS
l11..	ALUMINIUM ACETATE [EAR]
l12..	ACETIC ACID [EAR]
l2...	OTITIS EXTERNA ANTI - INFLAMMATORY PREPARATIONS
l21..	BETAMETHASONE SODIUM PHOSPHATE [EAR]
l22..	PREDNISOLONE SODIUM PHOSPHATE [EAR]
l3...	AURAL ANTI-INFECTIVE PREPARATIONS
l31..	CHLORAMPHENICOL [EAR]
l32..	CLIOQUINOL
l33..	CLOTRIMAZOLE [EAR]
l34..	*FRAMYCETIN SULPHATE [EAR]
l35..	GENTAMICIN [EAR]
l36..	NEOMYCIN SULFATE [EAR]
l37..	TETRACYCLINE HYDROCHLORIDE [EAR]
l4...	AURAL ANTI-INFECTIVE COMPOUNDS
l41..	AURAL ANTI-INFECTIVE COMPOUNDS
l5...	OTHER AURAL PREPARATIONS
l51..	OTHER AURAL PREPARATIONS
l6...	*OTITIS MEDIA [no drugs here]
l7...	REMOVAL OF EAR WAX
l71..	REMOVAL OF EAR WAX
l8...	NASAL ALLERGY DRUGS
l81..	BECLOMETASONE DIPROPIONATE [NOSE]
l82..	BETAMETHASONE SOD PHOSPHATE [NOSE]
l83..	BUDESONIDE [NOSE]
l84..	FLUNISOLIDE
l85..	SODIUM CROMOGLICATE [NOSE]
l86..	IPRATROPIUM BROMIDE [2]
l87..	AZELASTINE HYDROCHLORIDE
l88..	FLUTICASONE [NOSE]
l89..	NEDOCROMIL SODIUM [NOSE]
l8A..	LEVOCABASTINE HYDROCHLORIDE [NOSE]
l8B..	TRIAMCINOLONE ACETONIDE [NOSE]
l8C..	TRAMAZOLINE HYDROCHLORIDE+DEXAMETHASONE ISONICOTINATE

l8D..	MOMETASONE FUROATE MONOHYDRATE [NOSE]
l8E..	AZELASTINE HYDROCHLORIDE+FLUTICASONE PROPIONATE
l9...	TOPICAL NASAL DECONGESTANTS
l91..	EPHEDRINE HYDROCHLORIDE [NOSE]
l92..	OXYMETAZOLINE HYDROCHLORIDE
l93..	PHENYLEPHRINE HYDROCHLORIDE [NOSE]
l94..	XYLOMETAZOLINE HYDROCHLORIDE
l95..	*COMPOUND NASAL DECONGESTANTS
la...	ANTI-INFECTIVE NASAL PREPS.
lA...	OTHER NASAL PREPARATIONS
la1..	ANTI-INFECTIVE NASAL PREPS.
lA1..	SODIUM CHLORIDE [NOSE]
lA2..	GLUCOSE+GLYERCOL
lA3..	CELLULOSE
lA4..	SESAME OIL
lb...	APHTHOUS MOUTH ULCER DRUGS
lb1..	BENZYDAMINE HYDROCHLORIDE
lb2..	CARBENOXOLONE SODIUM [MOUTH]
lb3..	CARMELLOSE SODIUM
lb4..	CORTICOSTEROIDS FOR OROPHARYNX
lb5..	LOCAL ANAESTHETICS-OROPHARYNX
lb6..	SALICYLATES - FOR OROPHARYNX
lb7..	TETRACYCLINE FOR OROPHARYNX
lc...	OROPHARYNGEAL ANTI-INFECTIVES
lc1..	AMPHOTERICIN [OROPHARYNGEAL]
lc2..	DEQUALINIUM CHLORIDE
lc3..	*IDOXURIDINE [MOUTH- no preps]
lc4..	MICONAZOLE [OROPHARYNGEAL]
lc5..	*NATAMYCIN [MOUTH-no preps]
lc6..	NYSTATIN [MOUTH]
lc7..	*POLYNOXYLIN
lc8..	TETRACYCLINE [mouth-no drugs here]
lc9..	MINOCYCLINE HYDROCHLORIDE
lcA..	METRONIDAZOLE BENZOATE [OROPHARYNG]
ld...	ANTISEPTIC LOZENGES AND SPRAYS
ld1..	ANTISEPTIC LOZENGES AND SPRAYS
le...	MOUTH-WASHES/GARGLES/DENTIFRICES
le1..	CETYLPYRIDINIUM CHLORIDE
le2..	CHLORHEXIDINE GLUCONATE
le3..	HEXETIDINE
le4..	HYDROGEN PEROXIDE [MOUTH]
le5..	PHENOL [MOUTH]
le6..	POVIDONE-IODINE [MOUTH]
le7..	SODIUM CHLORIDE [MOUTH]
le8..	SODIUM PERBORATE

le9..	THYMOL
leA..	POVIDONE [MOUTH]
leB..	OTHER MOUTHWASH
leC..	OTHER DENTIFRICES
lf...	OTHER OROPHARYNGEAL PREPARATIONS
lf1..	OTHER OROPHARYNGEAL PREPARATIONS
lf2..	PILOCARPINE HYDROCHLORIDE [OROPHARYNGEAL]
lf3..	FLURBIPROFEN [OROPHARYNGEAL]
lf4..	HYALURONIC ACID
m....	SKIN DRUGS
m1...	VEHICLES AND DILUENTS
m11..	VEHICLES + DILUENTS : CREAMS
m12..	VEHICLES+DILUENTS : OINTMENTS
m13..	VEHICLES + DILUENTS : LOTIONS - [no drugs here]
m14..	VEHICLES+DILUENTS:SHAKE LOTION - [no drugs here]
m15..	VEHICLES + DILUENTS : PASTES
m16..	VEHICLES+DILUENTS:APPLICATIONS - [no drugs here]
m17..	VEHICLES+DILUENTS:COLLODIONS
m18..	VEHICLES + DILUENTS: LINIMENTS - [no drugs here]
m19..	VEHICLES + DILUENTS : PAINTS - [no drugs here]
m2...	EMOLLIENTS + BARRIER PREPARATIONS
m21..	EMOLLIENTS/BARRIER CREAMS: A-E
m22..	EMOLLIENTS/BARRIER CREAMS: F-O
m23..	EMOLLIENTS/BARRIER CREAMS: P-Z
m24..	UREA CREAMS AND LOTIONS
m25..	EMOLLIENT BATH ADDITIVES
m26..	DUSTING POWDERS
m27..	ANTISEPTIC EMOLLIENTS
m28..	EMOLLIENTS/BARRIER CREAMS:A-E (1)
m29..	EMOLLIENT BATH ADDITIVES (2)
m3...	LOCAL ANAESTHETIC/ANTIPRURITIC
m31..	CALAMINE
m32..	CROTAMITON [ANTIPRURITIC]
m33..	LOCAL ANAESTHETICS [SKIN]
m34..	TOPICAL ANTIHISTAMINES
m35..	DOXEPIN HYDROCHLORIDE [ANTIPRURITIC]
m36..	MENTHOL IN AQUEOUS CREAM
m37..	TOPICAL SKIN COOLANTS
m4...	TOPICAL CORTICOSTEROIDS
m41..	HYDROCORTISONE CREAMS
m42..	HYDROCORTISONE OINTMENTS
m43..	HYDROCORTISONE LOTIONS/DRESSNG
m44..	HYDROCORTISONE COMPOUND PREPARATIONS
m45..	HYDROCORTISONE+ANTIMICROBIALS
m46..	ALCLOMETASONE DIPROPIONATE

m47..	BECLOMETASONE DIPROP [SKIN]
m48..	BETAMETHASONE ESTERS
m49..	BETAMETHASONE + ANTIMICROBIALS
m4a..	CLOBETASOL PROPIONATE
m4b..	CLOBETASONE BUTYRATE [SKIN]
m4c..	*DESONIDE
m4d..	DESOXIMETASONE
m4e..	DIFLUCORTOLONE VALERATE
m4f..	FLUCLOROLONE ACETONIDE
m4g..	FLUOCINOLONE ACETONIDE
m4h..	FLUOCINONIDE
m4i..	FLUOCORTOLONE
m4j..	FLUDROXYCORTIDE
m4k..	HALCINONIDE
m4l..	HYDROCORTISONE BUTYRATE
m4m..	METHYLPREDNISOLONE ACETATE
m4n..	TRIAMCINOLONE ACETONIDE [SKIN]
m4o..	BUDESONIDE TOPICAL
m4p..	HYDROCORTISONE ACETATE [TOPICAL]
m4q..	MOMETASONE FUROATE [TOPICAL]
m4r..	FLUTICASONE PROPIONATE[SKIN]
m4s..	MOMETASONE FUROATE MONOHYDRATE
m5...	PSORIASIS AND ECZEMA PREPARATIONS
m51..	COAL TAR
m52..	*BUFEXAMAC
m53..	DITHRANOL
m54..	DITHRANOL TRIACETATE
m55..	*ETRETINATE
m56..	ICHTHAMMOL
m57..	SALICYLIC ACID [PSORIASIS]
m58..	GAMOLENIC ACID
m59..	CALCIPOTRIOL
m5A..	ACITRETIN
m5B..	TACALCITOL
m5C..	TAZAROTENE
m5D..	COAL TAR [2]
m5E..	TACROLIMUS [SKIN]
m5F..	PIMECROLIMUS
m5G..	PINE TAR
m5H..	ALITRETINOIN
m5z..	DITHRANOL [GENERIC ADDITIONS]
m6...	ACNE - TOPICAL PREPARATIONS
m61..	ABRASIVE AGENTS - ACNE
m62..	BENZOYL PEROXIDE [ACNE]
m63..	SULFUR

Wilkinson T, *et al. J Epidemiol Community Health* 2022; 76:223–229. doi: 10.1136/jech-2021-217090

m64..	*TETRACYCLINES [SKIN]
m65..	TRETINOIN [SKIN]
m66..	CORTICOSTEROIDS [SKIN]
m67..	ERYTHROMYCIN [SKIN]
m68..	CLINDAMYCIN [SKIN]
m69..	ISOTRETINOIN [TOPICAL]
m6A..	AZELAIC ACID
m6B..	SALICYLIC ACID [ACNE]
m6C..	NICOTINAMIDE [SKIN]
m6D..	ADAPALENE
m6z..	BENZOYL PEROXIDE [GENERIC ADDITIONS]
m7...	ACNE - ORAL PREPARATIONS
m71..	CYPROTERONE ACETATE [ACNE]
m72..	ISOTRETINOIN
m8...	WARTS AND CALLUSES
m81..	SALICYLIC ACID [CALLOSITY]
m82..	BROMINE COMPLEXES
m83..	FORMALDEHYDE
m84..	GLUTARALDEHYDE
m85..	PODOPHYLLUM RESIN
m86..	PODOPHYLLOTOXIN
m87..	DIMETHYL-ETHER PROPANE
m88..	SILVER NITRATE [WARTS]
m89..	POTASSIUM HYDROXIDE
m9...	SUNSCREENING PREPARATIONS
m91..	SUNSCREENING PREPARATIONS A-Z
m92..	SUNSCREENING PREPRATIONS A-Z [2]
ma...	CAMOUFLAGING PREPARATIONS
ma1..	CAMOUFLAGING PREPARATIONS A-Z
ma3..	CAMOUFLAGING PREPARATIONS[2] A-Z
ma4..	CAMOUFLAGING PREPARATIONS[3] A-Z
mb...	SCALP PREPARATIONS
mb1..	SHAMPOOS
mb2..	SCALP APPLICATIONS
mb3..	MINOXIDIL [ANDROGEN ALOPECIA]
mb4..	LITHIUM SUCCINATE [TOPICAL]
mb5..	SHAMPOOS [2]
mc...	ANTIBACTERIAL TOPICAL PREPARATIONS
mc1..	CHLORAMPHENICOL [SKIN]
mc2..	COLISTIN SULFATE
mc3..	FRAMYCETIN SULFATE [SKIN]
mc4..	*MAFENIDE
mc5..	MUPIROCIN
mc6..	NEOMYCIN SULFATE [SKIN]
mc7..	*NITROFURAZONE

mc8..	POLYMYXIN B SULFATE [SKIN]
mc9..	SILVER SULFADIAZINE
mcA..	RETAPAMULIN
md...	ANTIBACT. - SYSTEMIC USE ALSO
md1..	CHLORTETRACYCLINE HYDROCHLORIDE [SKIN]
md2..	FUSIDIC ACID
md3..	GENTAMICIN [SKIN]
md4..	TETRACYCLINE HYDROCHLORIDE [SKIN]
me...	ANTIFUNGAL PREPARATIONS
me1..	*AMPHOTERICIN [SKIN]
me2..	BENZOIC ACID
me3..	BENZOYL PEROXIDE [ANTIFUNGAL]
me4..	CLOTRIMAZOLE [SKIN]
me5..	ECONAZOLE NITRATE [SKIN]
me6..	KETOCONAZOLE [SKIN]
me7..	MICONAZOLE NITRATE [SKIN]
me8..	*NATAMYCIN [SKIN]
me9..	*NITROPHENOL
mea..	NYSTATIN [SKIN]
meb..	SALICYLIC ACID [ANTIFUNGAL]
mec..	SULCONAZOLE NITRATE
med..	TOLNAFTATE
mee..	UNDECENOATES
mef..	TIOCONAZOLE
meg..	METRONIDAZOLE [SKIN]
meh..	AMOROLFINE HYDROCHLORIDE
mej..	TERBINAFINE HYDROCHLORIDE [2]
mek..	BIFONAZOLE (SKIN)
mel..	FLUTRIMAZOLE
mf...	ANTIVIRAL PREPARATIONS
mf1..	ACICLOVIR [SKIN]
mf2..	IDOXURIDINE+DIMETHYLSULFOXIDE
mf3..	PENCICLOVIR [SKIN]
mg...	PARASITICIDAL PREPARATIONS
mg1..	BENZYL BENZOATE
mg2..	CARBARYL
mg3..	CROTAMITON [ANTIPARASITIC - no drugs here]
mg4..	LINDANE
mg5..	MALATHION
mg6..	MONOSULFIRAM
mg7..	PHENOTHRIN
mg8..	PERMETHRIN
mh...	MINOR SKIN INFECTION PREPARATIONS
mh1..	MINOR SKIN INFECTION PREPARATIONS A-Z
mi...	DISINFECTING/CLEANSING AGENTS

mi1..	ALCOHOL
mi2..	ALUMINIUM ACETATE
mi3..	BENZALKONIUM CHLORIDE
mi4..	CETRIMIDE
mi5..	CHLORHEXIDINE A-H
mi6..	CHLORHEXIDINE I-Z
mi7..	CHLORINATED SOLUTIONS
mi8..	CHLOROXYLENOL
mi9..	CRYSTAL VIOLET
mia..	HEXACHLOROPHENE
mib..	HYDROGEN PEROXIDE [SKIN]
mic..	IODINE COMPOUNDS
mid..	POTASSIUM PERMANGANATE
mie..	SILVER NITRATE [DISINFECTANT]
mif..	SODIUM CHLORIDE [SKIN]
mig..	SOFT SOAP
mih..	TRICLOSAN
mii..	ZINC SULFATE [SKIN]
mij..	WATER FOR IRRIGATION
mik..	COMBINATION WOUND CLEANSERS
mil..	OCTENIDINE
mim..	POLIHEXANIDE
mj...	DESLOUGHING AGENTS
mj1..	ASERBINE
mk...	SOAP SUBSTITUTES - [no drugs here]
ml...	ANTIPERSPIRANTS
ml1..	ALUMINIUM CHLORIDE
ml2..	GLYCOPYRROLATE
mn...	WOUND/ULCER PREPARATIONS
mn1..	CLEANSING PREPARATIONS
mn2..	DRESSINGS
mn3..	TISSUE ADHESIVE
mn4..	BECAPLERMIN
mn5..	PALIFERMIN
mn6..	CLEANSING PREPARATIONS(2)
mn7..	DRESSINGS(2)
mn8..	PHYSICAL DEBRIDEMENT DEVICE
mn9..	DRESSINGS (3)
mo...	CIRCULATORY TOPICAL PREPARATIONS
mo1..	CIRCULATORY TOPICAL PREPARATIONS A-Z
mp...	ELECTRODE+ULTRASOUND GELS AND CREAMS
mp1..	ELECTRODE GEL
mp2..	ULTRASOUND COUPLING GEL
mp3..	ELECTRODE CREAM
mq...	SKIN BLEACHING PREPARATIONS

Wilkinson T, *et al. J Epidemiol Community Health* 2022; 76:223–229. doi: 10.1136/jech-2021-217090

mq1..	HYDROQUINONE
mr...	PHARMACEUTICAL SOLVENTS
mr1..	SOLVENT ETHER
mr2..	ACETONE PRODUCT
ms...	NAIL BED PREPARATIONS
ms1..	PHENOL [PODIATRY]
mt...	ACTINIC KERATOSES PREPARATIONS
mt1..	DICLOFENAC SODIUM [ACTINIC KERATOSIS]
mt2..	INGENOL MEBUTATE
mu...	HIRSUTISM PREPARATIONS
mu1..	EFLORNITHINE
mv...	OTHER DERMATOLOGICAL PREPARATIONS
mv1..	IVERMECTIN
n....	IMMUNOLOGY DRUGS AND VACCINES
n1...	ACTIVE IMMUNITY [no drugs here]
n2...	PASSIVE IMMUNITY [no drugs here]
n3...	STORAGE AND USE OF VACCINES [no drugs here]
n4...	VACCINES AND ANTISERA
n41..	ANTHRAX VACCINE
n42..	BCG VACCINES
n43..	BOTULISM ANTITOXIN
n44..	CHOLERA VACCINE
n45..	DIPHTHERIA VACCINES
n46..	HEPATITIS B VACCINE
n47..	INFLUENZA VACCINES
n48..	*MEASLES VACCINE
n49..	MUMPS VACCINE
n4a..	PERTUSSIS VACCINE
n4b..	PNEUMOCOCCAL VACCINE
n4c..	POLIOMYELITIS VACCINE
n4d..	RABIES VACCINE
n4e..	RUBELLA VACCINE
n4f..	SMALLPOX VACCINE
n4g..	TETANUS VACCINES
n4h..	TYPHOID VACCINE
n4i..	*TYPHUS VACCINE
n4j..	YELLOW FEVER VACCINE
n4k..	MEASLES/MUMPS/RUBELLA VACCINE
n4l..	MENINGITIS VACCINE
n4m..	HEPATITIS A VACCINE
n4n..	HAEMOPHILUS INFLUENZAE Type b (Hib) VACCINE
n4o..	*MEASLES+RUBELLA VACCINE
n4p..	HAEMOPHILUS INFLUENZAE TYPE b (HIB)/DIPHTHERIA/TETANUS/PERTUSSIS VACCINE
n4q..	JAPANESE B ENCEPHALITIS VACCINE

n4r..	TICK-BORNE ENCEPHALITIS VACCINE
n4s..	HEPATITIS A+B VACCINE
n4t..	PLAGUE VACCINE
n4u..	HEPATITIS A+TYPHOID VACCINE
n4v..	VARICELLA-ZOSTER VACCINE
n4w..	ROTAVIRUS VACCINE
n4x..	HAEMOPHILUS INFLUENZAE type b + MENINGITIS C VACCINE
n4y..	HAEMOPHILUS INFLUENZAE type B/DIPHTHERIA/TETANUS/PERTUSSIS/POLIO V
n4z..	HUMAN PAPILLOMAVIRUS VACCINE
n5...	IMMUNOGLOBULINS
n51..	NORMAL IMMUNOGLOBULIN
n52..	SPECIFIC IMMUNOGLOBULINS
n53..	ANTI-D (RH) IMMUNOGLOBULIN
n54..	HUMAN TICK-BORNE ENCEPHALITIS IMMUNOGLOBULIN
n55..	NORMAL IMMUNOGLOBULIN(2)
n56..	NORMAL IMMUNOGLOBULIN(3)
n6...	VACCINATION PROGRAMMES -[no drugs here]
n7...	INTERNATIONAL TRAVEL - [no drugs here]
n8...	HUMAN MONOCLONAL IgM ANTIBODY
n81..	*HA-1A

Codes used to identify all-cause dementia and Alzheimer's disease cases

All-cause dementia - ICD-10

Description	Code
Dementia in Alzheimer's disease	F00
Dementia in Alzheimer's disease with early onset	F00.0
Dementia in Alzheimer's disease with late onset	F00.1
Dementia in Alzheimer's disease, atypical or mixed type	F00.2
Dementia in Alzheimer's disease, unspecified	F00.9
Alzheimer's disease	G30
Alzheimer's disease with early onset	G30.0
Alzheimer's disease with late onset	G30.1
Other Alzheimer's disease	G30.8
Alzheimer's disease unspecified	G30.9
Vascular dementia	F01
Vascular dementia of acute onset	F01.0
Multi-infarct dementia	F01.1
Subcortical vascular dementia	F01.2
Mixed cortical and sub-cortical vascular dementia	F01.3
Other vascular dementia	F01.8
Vascular dementia, unspecified	F01.9
Binswanger's disease	I67.3
Dementia in Picks disease	F02.0
Circumscribed brain atrophy	G31.0
Sporadic Creutzfeldt-Jakob disease	A81.0
Dementia in Creutzfeldt-Jacob disease	F02.1
Dementia in Huntington's disease	F02.2
Dementia in Parkinson's disease	F02.3
Dementia in HIV disease	F02.4
Mental and behavioural disorders due to use of alcohol - amnesic syndrome	F10.6
Dementia in other diseases classified elsewhere	F02
Dementia in other specified diseases classified elsewhere	F02.8
Unspecified dementia	F03
Delirium superimposed on dementia	F05.1
Senile degeneration of brain	G31.1
Other specified degenerative diseases of nervous system	G31.8

All-cause dementia - Read version 2

Description	Code
[X] Dementia in Alzheimer's disease	Eu00.
[X]Dementia in Alzheimer's disease with early onset	Eu000
[X]Dementia in Alzheimer's disease with late onset	Eu001
[X]Dementia in Alzheimer's disease, atypical or mixed type	Eu002
[X]Dementia in Alzheimer's disease, unspecified	Eu00z

Alzheimer’s disease	F110.
Alzheimer’s disease with early onset	F1100
Alzheimer’s disease with late onset	F1101
Senile degeneration of brain	F112.
[X] Other Alzheimer's disease	Fyu30
Multi-infarct dementia	E004.
Uncomplicated arteriosclerotic dementia	E0040
Arteriosclerotic dementia with delirium	E0041
Arteriosclerotic dementia with paranoia	E0042
Arteriosclerotic dementia with depression	E0043
Arteriosclerotic dementia NOS	E004z
[X]Vascular dementia	Eu01.
[X]Vascular dementia of acute onset	Eu010
[X]Multi-infarct dementia	Eu011
[X]Other vascular dementia	Eu01y
[X]Vascular dementia, unspecified	Eu01z
Cerebral degeneration due to cerebrovascular disease	F11x2
Binswanger's disease	F21y2
[X] Lewy body dementia	Eu025
Lewy body disease	F116.
[X] Dementia in Picks disease	Eu020
Pick's disease	F111.
Frontotemporal degeneration	F118.
Jakob-Creutzfeldt disease	A411.
Sporadic Creutzfeldt-Jakob disease	A4110
Alcoholic dementia, NOS	E012.
Dementia in conditions EC	E041.
[X] Dementia in other diseases classified elsewhere	Eu02.
[X] Dementia in Creutzfeldt-Jacob disease	Eu021
[X] Dementia in Huntington’s disease	Eu022
[X] Dementia in Parkinson’s disease	Eu023
[X] Dementia in HIV disease	Eu024
[X]Dementia in other specified diseases classified elsewhere	Eu02y
[X]Mental and behavioural disorders due to use of alcohol: amnesic syndrome	Eu106
[X]Mental and behavioural disorders due to use of alcohol: residual and late-onset psychotic disorder	Eu107
Cerebral degeneration due to Jacob-Creutzfeldt disease	F11x7
Cerebral degeneration due to Parkinson’s disease	F11x9
Corticobasal degeneration	F11y2
H/O: dementia	1461.
Assessment of psychotic and behavioural symptoms of dementia	38C13
GDS level 4 - moderate cognitive decline	3AE3.
GDS level 5 - moderately severe cognitive decline	3AE4.
GDS level 6 - severe cognitive decline	3AE5.
GDS level 7 - very severe cognitive decline	3AE6.

Wilkinson T, *et al. J Epidemiol Community Health* 2022; 76:223–229. doi: 10.1136/jech-2021-217090

Dementia monitoring	66h..
Dementia annual review	6AB..
Dementia medication review	8BM02
Shared care – prescribing drug for dementia	8BM50
Shared care – prescribing drug for dementia declined	8BM60
Antipsyc drug therapy dementia	8BPa.
Dementia advance care plan	8CMe0
Review of dementia advance care plan	8CMG2
Dementia care plan	8CMZ.
Dementia care plan agreed	8CMZ0
Dementia care plan reviewed	8CMZ1
Dementia care plan declined	8CMZ2
Dementia care plan review declined	8CMZ3
Dementia advance care plan agreed	8CSA.
Referral to dementia care advisor	8Hla.
Dementia adv care plan declnd	8IAe0
Dementia advance care plan review declined	8IAe2
Exception reporting: dementia quality indicators	9hD..
Excepted from dementia quality indicators: patient unsuitable	9hD0.
Excepted from dementia quality indicators: informed dissent	9hD1.
Dementia monitoring administration	9Ou..
Dementia monitoring first letter	9Ou1.
Dementia monitoring second letter	9Ou2.
Dementia monitoring third letter	9Ou3.
Dementia monitoring verbal invite	9Ou4.
Dementia monitoring telephone invite	9Ou5.
Senile and presenile organic psychotic condition	E00..
Uncomplicated senile dementia	E000.
Pre-senile dementia	E001.
Uncomplicated pre-senile dementia	E0010
Pre-senile dementia with delirium	E0011
Pre-senile dementia with paranoia	E0012
Pre-senile dementia with depression	E0013
Pre-senile dementia NOS	E001z
Senile dementia with depressive or paranoid features	E002.
Senile dementia with paranoia	E0020
Senile dementia with depression	E0021
Senile dementia with depressive or paranoid features NOS	E002z
Senile dementia with delirium	E003.
Drug induced dementia	E02y1
[X]Sub-cortical vascular dementia	Eu012
[X]Mixed cortical and sub-cortical vascular dementia	Eu013
[X] Unspecified dementia	Eu02z
[X] Delirium superimposed on dementia	Eu041

Alzheimer's disease – ICD-10

Description	Code
Dementia in Alzheimer's disease	F00
Dementia in Alzheimer's disease with early onset	F00.0
Dementia in Alzheimer's disease with late onset	F00.1
Dementia in Alzheimer's disease, atypical or mixed type	F00.2
Dementia in Alzheimer's disease, unspecified	F00.9
Alzheimer’s disease	G30
Alzheimer’s disease with early onset	G30.0
Alzheimer’s disease with late onset	G30.1
Other Alzheimer's disease	G30.8
Alzheimer's disease unspecified	G30.9

Alzheimer’s disease – Read V2

Description	Code
[X] Dementia in Alzheimer's disease	Eu00.
[X]Dementia in Alzheimer's disease with early onset	Eu000
[X]Dementia in Alzheimer's disease with late onset	Eu001
[X]Dementia in Alzheimer's disease, atypical or mixed type	Eu002
[X]Dementia in Alzheimer's disease, unspecified	Eu00z
Alzheimer’s disease	F110.
Alzheimer’s disease with early onset	F1100
Alzheimer’s disease with late onset	F1101
Senile degeneration of brain	F112.
[X] Other Alzheimer's disease	Fyu30

Further code lists used to derive the Secure Anonymised Information Linkage databank Dementia electronic Cohort (SAIL-DeC) are available at: https://datashare.is.ed.ac.uk/handle/10283/3268.

The construction of SAIL-DeC is outlined in Schnier C, Wilkinson T et al. *Cohort profile: The Secure Anonymised Information Linkage databank Dementia e-cohort (SAIL-DeC)*. International Journal of Population Data Science. 2020 Feb 25;5(1).

Supplementary Results: Medications predicted a priori to be associated with dementia (Group 1)

Neurodegenerative diseases

All drugs in the 'Drugs used in parkinsonism and related disorders' (BNF section 4.9) section were associated with dementia (HR range 2.86-8.55).

Cardiovascular diseases

Many medications prescribed for cardiovascular diseases were associated with dementia, across multiple BNF sections. This included drugs such as: digoxin (HR 1.67); diuretics, such as furosemide (HR 1.54) and spironolactone (HR 1.45), beta-adrenoreceptor blocking drugs, such as propranolol (HR 1.29); nitrates; anticoagulants such as warfarin (HR 1.34) and rivaroxaban (HR 2.08); antiplatelets, such as aspirin (HR 1.60), dipyridamole (HR 2.21) and clopidogrel (HR 1.70) and statins.

Diabetes

Almost all drugs for diabetes were associated or tentatively associated with an increased dementia incidence. Most of the medications that were not associated had similar hazard ratios, but with small numbers of cases exposed.

Depression

Lithium (BNF section 4.2 - Drugs used in psychoses and related disorders) was associated with dementia with a large effect size (HR 3.59).

Among antidepressants (BNF section 4.3 - Antidepressant drugs), paroxetine, venlafaxine, citalopram, reboxetine, mirtazapine and escitalopram were all associated with dementia (HR range 1.61-2.95) with nefazodone tentatively associated (HR 1.73). Other drugs in this section had similar HRs, but with small numbers of cases exposed.

Symptoms of complications of dementia

All drugs in the section 'Drugs used in psychoses and related disorders' (BNF section 4.2) were associated with dementia, with large effect sizes (HR range 2.95-7.32), except for levomepromazine (also used as an antiemetic), which had few cases exposed (HR 1.91).

Many medications under sections such as 'fluids and electrolytes', 'oral nutrition', 'minerals' and 'vitamins' were associated with dementia. Of note, thiamine and 'vitamin B complex', often prescribed in in alcohol-dependence syndrome, had very large hazard ratios (HR 5.64 and 2.94 respectively).
